# Phylogenetic relationship between Australian *Fusarium oxysporum* isolates and resolving the species complex using the multispecies coalescent model

**DOI:** 10.1186/s12864-020-6640-y

**Published:** 2020-03-20

**Authors:** Saidi R. Achari, Jatinder Kaur, Quang Dinh, Ross Mann, Tim Sawbridge, Brett A. Summerell, Jacqueline Edwards

**Affiliations:** 1 AgriBio, Centre for AgriBioscience, Agriculture Victoria, Bundoora, Australia; 2 La Trobe University, Victoria, Australia; 3 Australian Institute of Botanical Science, Royal Botanic Gardens & Domain Trust, Sydney, Australia

**Keywords:** Phylogenomics, Taxonomy, Species delimitation, Sequencing, Recombination

## Abstract

**Background:**

The *Fusarium oxysporum* species complex (FOSC) is a ubiquitous group of fungal species readily isolated from agroecosystem and natural ecosystem soils which includes important plant and human pathogens. Genetic relatedness within the complex has been studied by sequencing either the genes or the barcoding gene regions within those genes. Phylogenetic analyses have demonstrated a great deal of diversity which is reflected in the differing number of clades identified: three, five and eight. Genetic limitation within the species in the complex has been studied through Genealogical Concordance Phylogenetic Species Recognition (GCPSR) analyses with varying number of phylogenetic ‘species’ identified ranging from two to 21. Such differing views have continued to confuse users of these taxonomies.

**Results:**

The phylogenetic relationships between Australian *F. oxysporum* isolates from both natural and agricultural ecosystems were determined using three datasets: whole genome, nuclear genes, and mitochondrial genome sequences. The phylogenies were concordant except for three isolates. There were three concordant clades from all the phylogenies suggesting similar evolutionary history for mitochondrial genome and nuclear genes for the isolates in these three clades. Applying a multispecies coalescent (MSC) model on the eight single copy nuclear protein coding genes from the nuclear gene dataset concluded that the three concordant clades correspond to three phylogenetic species within the FOSC. There was 100% posterior probability support for the formation of three species within the FOSC. This is the first report of using the MSC model to estimate species within the *F. oxysporum* species complex. The findings from this study were compared with previously published phylogenetics and species delimitation studies.

**Conclusion:**

Phylogenetic analyses using three different gene datasets from Australian *F. oxysporum* isolates have all supported the formation of three major clades which delineated into three species. Species 2 (Clade 3) may be called *F. oxysporum* as it contains the neotype for *F. oxysporum*.

## Introduction

The *Fusarium oxysporum* species complex (FOSC) is a group of economically important pathogenic [1] and putatively non-pathogenic strains which are morphologically similar but phylogenetically distinct [2, 3]. Members of this species complex display considerable ecological plasticity. Putatively non-pathogenic isolates are readily isolated from soil and roots of asymptomatic plants from both agricultural and natural ecosystems as endophytes [4] or as isolates which suppress soil-borne pathogens including pathogenic isolates of *F. oxysporum* [5, 6]. Furthermore, members of the FOSC are also associated with decayed plant material as saprophytes [7]. Plant pathogenic isolates are responsible for causing rots, damping-off and vascular wilts on a broad range of agronomically and horticulturally important crops [1]. There are also clinically important isolates which act as opportunistic pathogens causing infections in animals and immuno-suppressed humans [8, 9].

Despite having both mating-type genes, *F. oxysporum* has not been found to display a sexual life cycle. Historically, *F. oxysporum* taxonomy was based on the morphology of the asexual propagative structures. This led to a very broad species definition [10] which did not reflect the variability and genetic divergence within the species [11]. The intra-specific divergence was acknowledged by the concept of *forma specialis* (f.sp.) by Snyder et al. [12], which is a non-taxonomic entity. It is based on the pathogen-host specificity, although most isolates are putatively non-pathogenic soil inhabitants [13]. There are 106 well-characterised *formae speciales* (ff. spp.) [14] infecting more than 100 plant species [1, 15]. The current understanding of *F. oxysporum* as a species complex, comprising of many species and clades [16, 17], is far removed from the original broad species definition provided by Snyder et al. [10].

The advent of molecular sequencing technologies has enabled the study of phylogenetic relationships between the members of FOSC using multi-gene genealogies. Multi-gene genealogies use combinations of different mitochondrial and/or nuclear barcoding gene regions and have been increasingly used for molecular systematics. An early phylogenetic study by O’Donnell et al. [16] of 33 *F. oxysporum* isolates using two barcoding gene regions, translation elongation factor (*tef-1α*) and mitochondrial small subunit (*mtSSU rDNA*), divided the FOSC into three monophyletic clades. Laurence et al. [18] used the same barcoding loci and reported that 45 Australian *F. oxysporum* isolates from the natural ecosystem separated into five clades. Clade 4 comprised of only Australian isolates. More recently, Lombard et al. [19] identified eight clades within the FOSC using four barcoding gene regions, β-tubulin II (*tub2*), calmodulin (*cal*), the second largest subunit of DNA-dependent RNA polymerase II (*RPB2*) and *tef-1α*.

The uptake of whole genome sequencing resulting from low cost and high throughput of next-generation sequencing platforms has allowed the use of complete protein coding genes and complete mitochondrial (mt) genomes for phylogenetic analysis. The mt genome is present in high copy numbers which allows for mutations to occur without lethal impact [20]. This brings about an accelerated rate of evolution, making the mt genome a suitable region to study eukaryotic evolution [21]. Furthermore, gene loss appears to be irreversible [21] and the transfer of genetic material between or into the mt genome is thought to be limited [20]. Since the mt genome is relatively small, it can be studied in its entirety. The mitochondrial genome consists of two regions, a conserved region with relatively low levels of sequence variation and the large variable region (LV) [22] containing numerous sequence variations [23]. Sequence variations in this region are due to recombination events caused by parasexualism and this has resulted in three variant type sequences within the mitochondrial genome in FOSC [23]. Mt genome sequences have been used in molecular systematics and biodiversity studies of fungi at various taxonomic levels [24]. Three clades were identified in a phylogenetic analysis of the FOSC using the conserved region of the mt genome in combination with nine nuclear protein coding genes [23].

Molecular studies have demonstrated that genetic variations within the FOSC are not necessarily reflected in the ff. spp. concept. Polyphyly has further compounded the ff. spp. concept obscuring the genetic diversity of the isolates [16]. Initially, when the ff. spp. concept was attributed to phytopathogenic isolates of *F. oxysporum*, it was assumed that isolates which shared a host range would be more genetically similar than with isolates that did not share the same host range. Nucleic acid sequence analyses have shown that many of the ff. spp., previously assumed to be monophyletic, are polyphyletic or paraphyletic [16, 17, 25, 26]. Recent studies demonstrating that horizontal transfer of pathogenicity genes between isolates [27] counters the previous assumption that convergent evolution [28] has driven the polyphyletic phylogeny observed within the FOSC.

Identification and recognition of species within the FOSC is pivotal in areas of biology such as epidemiology (identification of novel pathogens) and evolutionary biology (describing diversification patterns) [29]. Although it is now accepted that the FOSC comprises a number of morphologically-similar cryptic species, the species boundaries and limits of genetic exchange are poorly defined, with different number of species predicted within the species complex in different studies. Two of these studies used Genealogical Concordance Phylogenetic Species Recognition (GCPSR) on different datasets for predicting the species boundaries. Laurence et al. [30] used barcoding regions of eight genes (*tef1-α*, *mtSSU*, largest subunit of DNA-dependent RNA polymerase II (*RPB1*), *RPB2*, nitrate reductase (*NIR*), phosphate permease (*PHO*), calmodulin (*cal*), ATP citrate lyase(*acl1*) and predicted two ‘species’, while Brankovics et al. [23] using the sequences of nine genes (γ-actin (*act*), cal, *RPB2*, *tef1-α*, *tef3*, 60Sribosomal protein L10 (*rpl10a*), topoisomerase I (*top1*), rDNA repeat and *tub2*) and the conserved part of the mitogenome predicted three ‘species’ which were concordant to the three clades in their phylogenetic analysis. Lombard et al. [19] identified 21 ‘species’ with no explanation of their model.

GCPSR in the above studies was implemented in two steps as defined by Dettman et al. [31] (i) identification of the independent evolutionary lineages (IEL) and (ii) exhaustive subdivision of isolates into phylogenetic species. IEL were identified based on concordance and non-discordance. Clades were concordant if they were supported by at least two single loci and compared to remove those that were discordant [23, 30]. IEL supported by at least half of the loci were kept as putative phylogenetic species. Each isolate had to be classified within a putative phylogenetic species. Exhaustive subdivision referred to collapsing of all the subclades of a clade when an isolate was grouped within that clade (putative phylogenetic species). This ensured that all phylogenetic species were monophyletic. The clades that remained were recognised as phylogenetic species [23, 30].

Species concepts in *F. oxysporum* have progressed from morphological to the use of multi-gene genealogies under GCPSR. The theoretical criteria for GCPSR developed by Taylor et al. [32] are based on Avise and Ball’s [33] genealogical concordance species concept. This states that recombination within a lineage will create conflict between gene trees and the transition from conflict to congruence represents the species limit [32]. However, there are other processes such as incomplete lineage sorting, horizontal gene transfer and population structure which could cause discordance between gene trees and species trees, masking true evolutionary relationships between closely related taxa [34]. Furthermore, the common practice of concatenating sequence data from multiple loci under GCPSR can lead to inaccuracies in species identification [35]. Alternatively, multispecies coalescent (MSC) models that incorporate gene tree uncertainty into species recognition may more accurately and objectively delimit species. Estimation of the speciation process using MSC model provides a more comprehensive speciation event as it recognises more gene discordant events than GCPSR. *Beast uses a multispecies based coalescent model for species delimitation using multi-locus sequence data [36]. Under this model, the gene trees are “embedded” in the species tree following stochastic coalescent processes while allowing for independent evolutionary processes in each genomic region [37]. A maximum clade credibility tree with posterior probability support for the nodes is computed from the gene trees. This is the species tree with each node denoting a species and the posterior probability support of the node showing the support for the denoted species to be called a species. This model allows testing of different scenarios for species assignments to find the best species fit for the lineage. One advantage of this model over other models is that it allows for the integration of knowledge from multi-gene trees into a single higher-level species tree during the delimitation process removing the constraint of specifying a guide tree for depicting species relationships [38].

MSC model-based species discrimination has previously been used for finding species boundaries in animal [39, 40] and plant taxa [41] and now it is being gradually adopted for resolving species complexes in fungal taxa. This model has been used by Stewart et al. [38] for species delimitation in a global population of the asexual fungus, *Alternaria alternata,* and by Liu et al. [42] for establishing species boundaries in the pathogenic fungal genus, *Colletotrichum*, which has a sexual state. Additionally, although a sexual state is unknown for *Fusarium oxysporum*, both mating-type genes are present, so the sexual cycle may have occurred at some point in its evolution.

### Objective

Previous studies identified considerable diversity within the FOSC using different datasets and methods. This has resulted in varying numbers of clades and species described. Therefore the objectives of this study were (i) to determine the phylogenetic relationships between Australian *F. oxysporum* isolates from natural and agroecosystems using three different datasets: the whole genome, the conserved region of the mitochondrial genome and eight informative nuclear genes (concatenated multi-loci), comparing them with previously published phylogenetic analyses, and (ii) to group the isolates into well supported lineages, i.e. ‘species’, using the multispecies coalescent model and to compare the species boundaries in the previous studies using the MSC model.

## Results

### Mitochondrial genome dataset

#### Mitochondrial genome sequences

The mitochondrial genome is divided into two parts based on sequence variations [22]. There is the conserved region of the mitochondrial genome and a region that shows higher levels of variation than any other parts of the mitogenome. This region is referred to as the large variable region (LV) which is located between *rnl* (mitochondrial *LSU rRNA* gene) and mitochondrially encoded NADH dehydrogenase 2 (*nad2*).

Sequences of the LV region of the mitochondrial genome were used to determine the mitochondrial genome variant type of the isolates. Variant 1 type mitochondrial genomes were the most dominant with 87 isolates, seven isolates belonged to Variant 2 and five isolates belonged to Variant 3. The average length of the LV region was significantly different (*p* < 0.05) between the mitochondrial genome variant types, with 11,515 bp for Variant 1, 17,738 bp for Variant 2 and 6065 bp for Variant 3 (Supplementary Figs. 1 and 2).

The average mitochondrial genome size also varied significantly (*p* < 0.05) between the variant types (Supplementary Table 1, Supplementary Figs. 2 and 3). Variant 1 was 44,455 bp, Variant 2 was 50,327 bp and Variant 3 was 37,148 bp.

The average size of the conserved region of the mitochondrial genome varied significantly between Variant 1 and Variant 3 only (Supplementary Figs. 2 and 4). The average size of the conserved region of Variant 1 was 32,940 bp, Variant 2 was 32,589 bp and Variant 3 was 31,082 bp. The sequences were very conserved between the variant types. They formed two clusters when compared against each other for percentage similarity using cd-hit-est (http://weizhong-lab.ucsd.edu/cdhit-web-server/cgi-bin/index.cgi) [43, 44]. Cluster one had sequences with more than 95% sequence identity, while cluster two had only 2 isolates (VPRI10358 and VPRI10405) with 92% sequence identity.

There were introns present in the following protein coding genes of the mitochondrial genome: mitochondrially encoded NADH dehydrogenase 5 (*nad5*) and mitochondrially encoded ATP synthase membrane subunit 6 gene (*atp6*) had one intron, and mitochondrially encoded cytochrome b (*cob*) had two introns (Supplementary Table 1). All the isolates had an intron of 1009 bp in *nad5*. Introns in *cob* ranged from 200 to 500 bp. There were 17 Variant 1, four Variant 2 and one Variant 3 isolates with an intron in position 1 of *cob* while only three isolates, two Variant 1 and one Variant 2, had an intron in position 2 of *cob* (Supplementary Table 1). Only three Variant 1 isolates had an intron in *atp6* which varied in length from 328 bp to 1238 bp (Supplementary Table 1).

#### Phylogenetic analysis

**Fig. 1:**
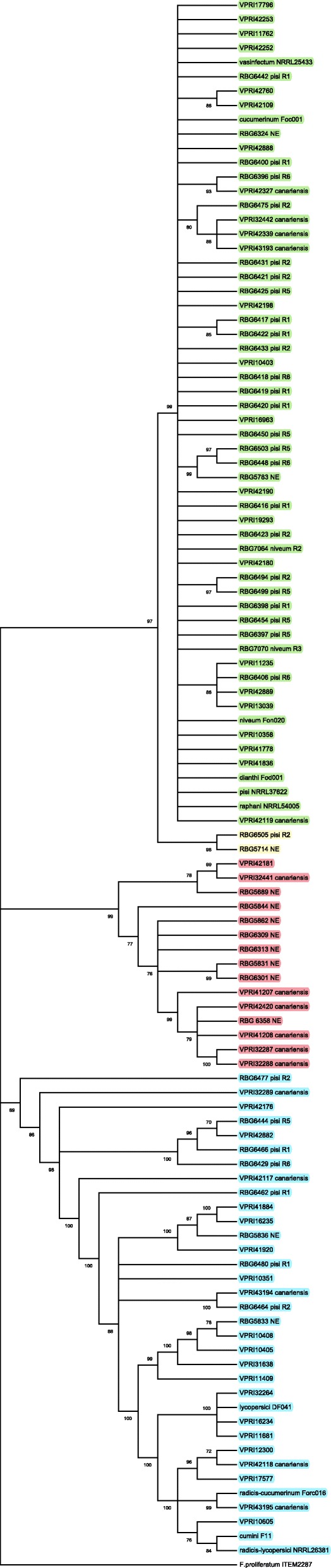
Maximum likelihood consensus tree with bootstrap node support of > 70% was inferred from conserved mitochondrial genome sequence of *Fusarium oxysporum* isolates used in the study and 10 reference isolates (NRRL25433, Foc001, Fon020, Fod001, NRRL37622, NRRL54005, DF041, Forc016, F11, NRRL26381) from Brankovics et al. [23] using MEGA X with 1000 bootstrap replications. The best nucleotide substitution model, HKY + G + I was used. The four phylogenetic clades identified within the FOSC are highlighted in different shades. Isolates coloured red, green, blue and yellow belong to Clades 1, 2, 3 and 4 respectively. The tree is rooted to *Fusarium proliferatum* (ITEM2287)

The phylogenetic relationship between the isolates was studied using the conserved and the LV regions of the mitochondrial genome. Four phylogenetic trees were constructed from the mitochondrial genome dataset: the conserved region (Fig. 1) and the LV region of the three mitochondrial genome variant types (Supplementary Figs. 5 and 6). The conserved region had 11,899 sites, of which 9785 were conserved sites and 2111 were variable sites. Of these variable sites, 1044 were parsimony informative sites.

The maximum likelihood (ML) tree generated from the conserved mitochondrial region formed four well-supported clades (Fig. 1). Clades 1, 2, 3 and 4 had 15, 52, 30 and 2 isolates respectively. Clade 1 consisted solely of *F. oxysporum* f.sp. *canariensis* (*Foc*) isolates and isolates from the natural ecosystems, plus one isolate (VPRI42181), isolated from a symptomatic tomato seedling (Supplementary Table 1). None of the *F. oxysporum* f.sp. *pisi* (*Fop*) isolates were in Clade 1. *Foc* isolates were also present in other clades. Clade 4 contained only two isolates RBG6505 and RBG5714. There was no correlation between the variant type and the clades in which they were grouped. Variant 1, 2 and 3 isolates were spread throughout the clades.

The LV region of Variant 1 type mitochondrial genome isolates had 5504 sites with 4806 conserved and 698 variable sites. Two hundred and forty-four of these variable sites were parsimony informative. The LV region of Variant 2 type mitochondrial genome isolates had 9713 sites of which 7540 sites were conserved and 2173 were variable, of which 271 were parsimony informative. The LV region of Variant 3 type mitochondrial genome isolates had 4209 sites with 3950 conserved and 259 variable sites. Out of these 259 sites, 214 were parsimony informative.

Phylogenetic analysis of the LV region of the Variant 1 type mitochondrial genome isolates resulted in three well-supported Clades (Supplementary Fig. 5). Clades 1 and 3 have many sub-clades while Clade 2 has only one isolate, VPRI42176. Phylogenetic analysis of the LV region of the Variant 2 type mitochondrial genome has four clades (Supplementary Fig. 6), while there are three clades in the LV region phylogeny of the Variant 3 type mitochondrial genome (Supplementary Fig. 5) with Clade 2 having a single isolate, RBG5844.

### Comparison to earlier studies

#### Brankovics’s

Comparison of the conserved region of the mitochondrial genome phylogenies from the current study with Brankovics et al. [23] study, showed that both phylogenies were congruent. The isolates from the three clades identified in Brankovics et al. [23] phylogeny and used as reference sequences grouped with the isolates of the respective clades in the conserved region of the mitochondrial genome phylogeny in the current study (Fig. 1).

### Whole genome dataset

#### Whole genome sequence

There were 6800 genes conserved across the genomes of all isolates including the outgroup (99 from this study and 10 from National Center for Biotechnology Information (NCBI GenBank)). These were determined by concatenating the protein sequences of orthologous protein groups created using Basic Local Alignment Search Tool-Protein (BLASTP NCBI) and TRIBE Markov Cluster (MCL) [45].

#### Phylogenetic analysis

**Fig. 2:**
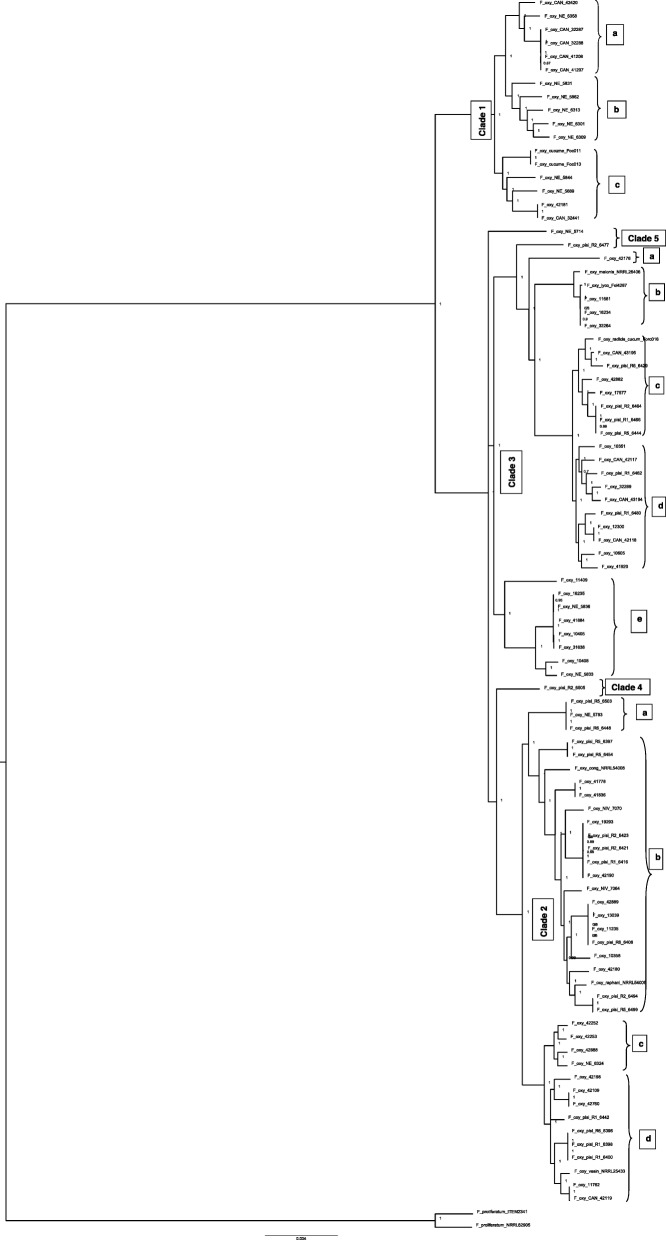
Phylogenetic analysis of whole genome of *Fusarium oxysporum* isolates used in the current study and eight reference isolates (NRRL25433, NRRL54005, NRRL54008, NRRL26406, Forc016, Fol4287, Foc013 and Foc011) included from GenBank using Roary: Pan Genome pipeline. Six thousand eight hundred genes were found to be conserved across all the isolates including the outgroup, *Fusarium proliferatum* (ITEM2341 and NRRL62905). The protein sequences for these genes were aligned using Multiple Alignment FAST Fourier Transform (MAFFT) [46] and then clustered into orthologous groups. A Maximum Likelihood tree was generated using FastTree [47] with the General Time Reversal (GTR) substitution model. FastTree used the Shimodaira-Hasegawa (SH) test for three alternate topologies for every split and each split was sampled 1000 times. The five phylogenetic clades and sub-clades identified within the FOSC are marked

The phylogenetic tree built with 6800 genes was used to study the relationship between the isolates from the natural and agroecosystems. The whole genome phylogeny gave a better resolution and population structure of the isolates than the phylogeny from the other two datasets. The whole genome phylogeny formed five well-supported clades with nodes having a local support value of 1 (100%) (ranges from 0 to 1) and separated by short branch lengths (Fig. 2). Clades 4 and 5 contained a single isolate each (RBG6505 and RBG5714 respectively). There was strong bootstrap support for the clades, with most of the nodes having 100% support. There were many highly supported sub-clades within the three major clades. Clade 1 had three highly supported sub-clades (a, b, c) consisting of 15 isolates and two reference isolates (*F. oxysporum* f.sp. *cucumerinum,* Foc011 and Foc013). Clade 2 had four very highly supported sub-clades (a, b, c, d) consisting of 52 isolates and three reference isolates [*F. oxysporum* f.sp. *conglutinans* (NRRL54008), *F. oxysporum* f.sp. *raphani* (NRRL54005) and *F. oxysporum* f.sp. *vasinfectum* (NRRL25433)]. Clade 3 had two single lineages (a) and four highly supported sub-clades (b, c, d, e). There were 30 isolates and three reference isolates [*F. oxysporum* f.sp. *melonis* (NRRL26406), *F. oxysporum* f.sp. *lycopersici* (Fol4287) and *F. oxysporum* f.sp. *radicis cucumerinum* (Forc016)]. Clade 1 isolates consisted mostly of those from the natural ecosystems (NE) and *F. oxysporum* f. sp. *canariensis* (CAN), while other clades contained isolates from the agroecosystem. There was no *F. oxysporum* f.sp. *pisi* isolate present in Clade 1.

### Nuclear gene dataset

#### Nuclear gene sequences

Eight single copy nuclear genes were concatenated with each gene having different number of informative sites (Table 1). The translation elongation factor 3 was the most informative gene while Calmodulin gene being the shortest gene had comparatively the least number of informative sites.

**Table 1:** The variability of the individual loci used for nuclear gene dataset phylogenetic analyses and species estimation

Locus	No. of sites	No. of parsimony informative sites
*tub2*	1590	35
*cal*	666	9
*mtSSU*	676	17
*RPB1*	1559	38
*RPB2*	2644	54
*tef1-α*	1228	32
*tef3*	3543	77
*Top1*	683	11

#### Phylogenetic analysis

**Fig. 3:**
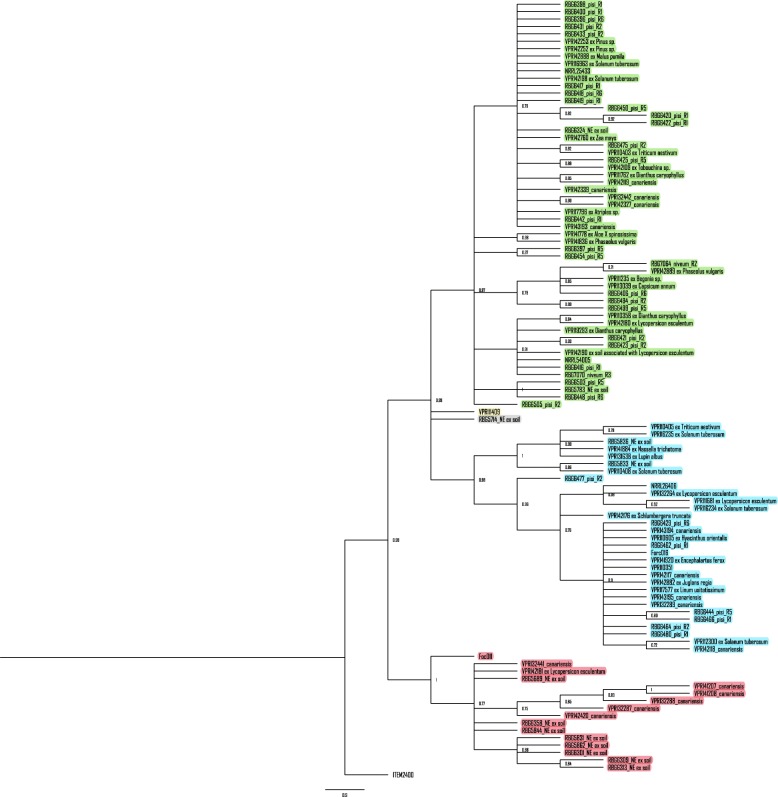
Maximum likelihood consensus tree with bootstrap node support of > 70% was inferred from the nuclear gene dataset (concatenated multi-locus DNA sequences of *tub2*, *cal*, *mtSSU*, *RPB1*, *RPB2*, *Tef1-α*, *TEF3* and *Top1*) of *Fusarium oxysporum* isolates used in the current study and five reference isolates (NRRL26406, NRRL54005, NRRL25433, Forc016 and Foc011) from Brankovics et al. [23] using MEGA X with 1000 bootstrap replications. The tree is rooted to *Fusarium proliferatum* (ITEM2400). The host species from which the Australian VPRI isolates were isolated from are shown on the tree. The five phylogenetic clades identified within the FOSC are highlighted in different shades. Isolates coloured red, green, blue, yellow and grey belong to Clades 1, 2, 3, 4 and 5 respectively

The phylogenetic analysis using the nuclear gene dataset (concatenated eight nuclear single copy genes) resulted in five well-supported clades. Clades 1, 2 and 3 have highly supported sub-clades. Clades 4 and 5 had a single isolate each (VPRI11409 and RBG5714 respectively). The ML tree topology was identical to the Bayesian inference (BI) tree topology, therefore, only the ML tree is presented (Fig. 3).

Individual analyses of the full sequences of the eight gene regions (*tub2*, *cal*, *mtSSU*, *RPB1*, *RPB2*, *tef1-α*, *tef3* and Topoisomerase I (*Top1*)) showed varying degrees of resolution for the formation of five clades. Apart from *cal* and *tef1-α*, all other genes had very high support (bootstrap value > 70%) and resolution for grouping of the isolates. *Top1* had high statistical support for the formation of Clade 1 and sub-clades in Clade 3 (a, b, c and d) (Fig. 2). Additionally, t*ub2* supported the formation of Clade 2b (Fig. 2). *mtSSU* supported the formation of sub-clade 3e (Fig. 2). *Tef3* supported the grouping of sub-clades 2b, 3b, 3c and 3e (Fig. 2). *RPB2* provided the best resolution with high statistical support for the formation of five clades, like the nuclear gene dataset phylogeny. The clade support from individual loci is representative of the number of informative sites per locus. *RPB2* and *tef3* had the highest number of informative sites, hence supported the formation of more clades, and conversely *cal* and *tef1-α* had the lowest number of informative sites, hence produced polytomies. Individual locus phylogeny trees are not presented.

Clades 1, 2 and 3 obtained from the phylogenies of the three datasets were congruent except for 3 isolates: RBG5714, VPRI11409 and RBG6505. Isolate RBG5714 present in Clade 5 of the nuclear gene dataset phylogeny is also in Clade 5 of the whole genome phylogeny but is present in Clade 4 of the conserved mitochondrial genome phylogeny. Isolate VPRI11409 present in Clade 4 of the nuclear gene dataset phylogeny is in Clade 3 of the whole genome phylogeny and conserved mitochondrial genome phylogeny. Isolate RBG6505 is present in Clade 2 of the nuclear gene dataset phylogeny, but in Clade 4 as a single isolate in the whole genome phylogeny and Clade 4 with isolate RBG5714 in the conserved mitochondrial genome phylogeny. Clade 1 in all phylogenies almost exclusively (exception of a single isolate, VPRI42181) consisted of isolates from natural ecosystems and *F. oxysporum* f.sp. *canariensis*. Isolates from agroecosystems were spread across the other clades.

### Comparison to earlier studies

#### Lombard’s, Brankovics’s, Laurence’s and O’Donnell’s datasets

The diversity of the complex was studied by comparing the clades in the current study with previously published phylogenies. The combined dataset from the current study and Lombard et al. [19] produced a phylogenetic tree with two clades (Supplementary Fig. 7). One clade consisted of a single isolate, and the rest were in the other clade. There were many sub-clades within this clade but with poor node support. The isolates from the current study grouped with isolates belonging to seven of the 21 ‘species’ from their study (Table 2). These ‘species’ were *F. odoratissimum*, *F. nirenbergiae*, *F*. *contaminatum*, *F*. *languescens*, *F. triseptatum*, *F. oxysporum* and *F. hoodiae.*

**Table 2:** Comparison of the clades from Lombard’s dataset phylogeny to the clades in the current study phylogenies

Lombard’s dataset phylogeny		Current study phylogenies		
Clade	species	Mitochondrial dataset	Whole genome dataset	Nuclear gene dataset
Clade 1	*F. odoratissimum*	Clade 1	Clade 1	Clade 1
Clade 4	*F. hoodiae*	Clade 4	Clade 5	Clade 5
Clade 5	*F. oxysporum* (neotype)	Sub-clade in Clade 3	Clade 3e	Sub-clade in Clade 3
Clade 5	*F. triseptatum*	Sub-clade in Clade 3	Clade 3a	Sub-clade in Clade 3
Clade 6	*F. languescens*	Sub-clade in Clade 3	Clade 3b	Sub-clade in Clade 3
Clade 7	*F. contaminatum*	Sub-clade in Clade 3	Clade 3a	Sub-clade in Clade 3
Clade 8	*F. nirenbergiae*	Sub-clade in Clade 3	Clades 3c and 3d	Sub-clade in Clade 3

The isolates representing the three clades in Brankovics et al. [48] study grouped with the isolates from the respective clades in the current study (Table 3), thus suggesting concordance between the phylogenetic trees obtained in their study to the current study.

**Table 3:** Summary of the relationship of the clades from the nuclear gene dataset from the current study to the previous studies

Current study	O’Donnell et al. (16)	Laurence et al. (30)	Brankovics et al. (23)
Clade 1	Clade 1	Clade 1	Clade 1
Clade 2	Clade 2	Clades 2 and 5	Clade 2
Clade 3	Clade 3	Clades 3 and 4	Clade 3
Clade 4	No match	No match	No match
Clade 5	No match	No match	No match

The combined dataset from the current study and Laurence et al. [30] produced a phylogenetic tree with two clades having many sub-clades (Supplementary Fig. 8). All Clade 1 isolates from the current study grouped with their isolates from Clade 1 (Table 3), which had been determined by GCPSR analysis to be phylogenetic ‘species’ 1. The remaining isolates from the current study grouped with their isolates from Clades 2–5 (Table 3), which were determined by GCPSR analysis to be phylogenetic ‘species’ 2.

The isolates representing the three clades from O’Donnell et al. [16] grouped with the isolates from the three clades in the current study (Table 3). None of the reference isolates from their study grouped with the isolates from Clades 4 and 5 from the current study.

#### Species tree estimation

**Fig. 4:**
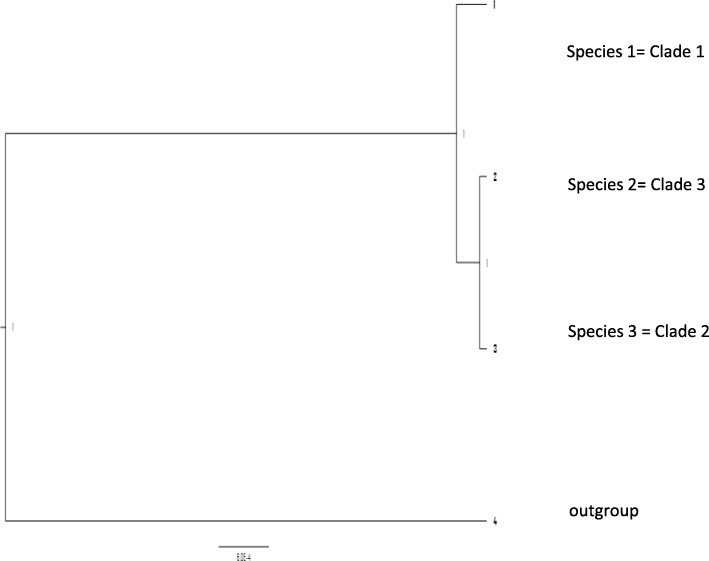
Species tree estimation of *Fusarium oxysporum* isolates using multispecies coalescent (MSC) model in *BEAST. Nuclear gene dataset with eight loci (*tef1-α*, tef3, RPB1, RPB2, cal, tub2, top1 and *mtSSU*) were used in the analysis. Numbers above branches indicate node support as posterior probabilities. The number of species correlates to the number of concordant clades (1, 2, and 3) in the phylogenies. Species denoted as 4 is the outgroup, *Fusarium proliferatum* (ITEM2400)

Applying the MSC model on the eight single copy nuclear genes (*tub2*, *ca*l, *RPB1*, *RPB2*, *tef1-α*, *tef3*, *top1* and *mtSSU*) resulted in the recognition of three species within the FOSC. The three concordant Clades (1, 2, and 3) in all the phylogenetic analyses (Figs. 1, 2, 3) were delimited as separate species with 100% posterior probability support at the nodes (Fig. 4). Species 1 in the tree contained isolates from Clade 1, species 2 contained isolates from Clade 3 and species 3 contained isolates from Clade 2. Species 2 (Clade 3) includes the neotype for *F. oxysporum* hence is the ‘true’ *F. oxysporum* species. The isolates RBG6505, RBG5714 and VPRI11409 which formed their own clades or grouped together in a clade in the 3 phylogenetics analyses were determined to belong to species 2. Four clades from conserved mitochondrial genome phylogeny, five clades from the nuclear gene dataset phylogeny and whole genome phylogeny and seven sub-clades within Clades 1, 2 and 3 from whole genome phylogeny were all tested as potential species, but the model gave very poor posterior probability support for the presence of four, five and seven species within the FOSC (data not shown).

### Comparison to earlier studies

Analyses of the datasets from previous studies, using the MSC model, showed that the results from the MSC model were concordant with the GCPSR, which was used for species delimitation in these studies. There was 100% posterior probability support for 2 ‘species’ within the FOSC from the Laurence et al. [30] dataset (not presented). There was also 100% posterior probability support for the presence of 3 ‘species’ within the FOSC from the Brankovics et al. [23] dataset (not presented).

Testing the Lombard et al. [19] dataset using the MSC model resulted in some well-supported nodes, depicted by species numbered 21, 17 and 9 (Supplementary Fig. 9). These were *F. veterinarium*, *F*. *oxysporum* and *F. foetens* respectively. *F. foetens* was used as an outgroup in their phylogenetic analyses. Species 17 contained the neotype for *F. oxysporum* isolated from potato tuber (*Solanum tuberosum*), so represents the ‘true’ *F. oxysporum*. There was support for the presence of 2 ‘species’ but not enough information within their dataset for supporting the other 19 ‘species’ that were identified in their study.

## Discussion

The aim of this project was to use whole genome, mitochondrial genome and nuclear (multi-locus) gene sequences to understand the phylogenetic relationship between Australian isolates of the *F. oxysporum* species complex and to group these isolates into well supported lineages, i.e. ‘species’. With over 100 well characterised ff. spp. [14] of *F. oxysporum*, Snyder and Hansen’s [49] definition of *F. oxysporum* has proven to be too broad to handle variability within the *F. oxysporum* population. The variability is not even reflected in the f. sp. concept as this naming concept is not a taxonomic entity.

Many studies have been carried out to understand the FOSC phylogeny using combinations of nuclear and mitochondrial barcoding gene regions [16, 18, 19, 25, 30] or genes [23] and effector genes [48, 50]. However, this is the first report of the FOSC phylogeny using the whole genome. Gene alignment and tree construction were based on 6800 genes. Whole genome phylogeny has provided a very robust phylogenetic framework, dividing the FOSC into five very well supported clades. The genome phylogeny has provided more resolution within the sub-clades providing more evolutionary information and a comprehensive population structure within these clades and the sub-clades.

Mitochondrial genome analyses revealed that there is recombination in the LV region of the mitochondrial genome which has resulted in three variant mitochondrial genome types present in Australia. Although *F. oxysporum* has an asexual lifecycle, recombination in the mitochondrial genome suggests that there is some mechanism which is allowing genetic exchange between the isolates. According to Brankovics et al. [23] this could be due to parasexualism. Variant 1 type mitochondrial genome was the most common (Supplementary Table S3), a finding similar to that of Brankovics et al. [23]. This finding has been supported by Xu et al. [51] who have reported that in ascomycetes there is no genetic factor that ensures uniparental *mtDNA* inheritance. There was no distinct pattern in the grouping of the variant types within the conserved region of the mitochondrial genome phylogeny. The three variant mitochondrial genome types were present in all three clades. The phylogenetic tree of Variant 2 mitochondrial genome type (Supplementary Fig. 6) is congruent with the conserved mitochondrial region phylogeny suggesting co-evolution of the conserved mitochondrial genome region and the LV region in these isolates. The conserved region of the mitochondrial genome phylogeny (Fig. 1) has four well-supported clades while in Brankovics et al. [23], there were only 3 clades. The nuclear gene dataset supported the formation of five well-supported clades (Fig. 4). Most isolates were in Clades 1, 2 and 3, with Clades 4 and 5 each made up of a single isolate. This shows that there is considerable genetic diversity in Australian *F. oxysporum* and this is in agreement with Laurence et al. [18] who had five clades within the FOSC from natural ecosystems when using the same gene barcoding region as O’Donnell et al. [16], whose isolates only grouped into three clades. Apart from Lombard et al. [19] and Laurence et al. [18] dataset, all other studies had three clades in the phylogenetic analyses. Greater genetic diversity within the Australian FOSC and Lombard et al. [19] datasets could be due to convergent evolution of isolates with their hosts [52]. Unique agricultural and ecological environments provide conducive environments for the pathogen to evolve. Another explanation for higher diversity, despite being an asexual pathogen, is the ability of the pathogen to transfer genes horizontally [53].

Different genes provide different topologies to a phylogenetic tree due to differences in their evolutionary history. Three basic sources of topological variations are mutation, lineage sorting and phylogenetic reconstruction artifacts [54]. Mutation and lineage sorting are natural sources of variation between genes. Mutation is caused stochastically and is more prevalent in short genes, preventing these genes in different species or isolates to truly reflect their phylogeny [54]. Lineage sorting is when diverging lineages maintain random ancestral polymorphism of a gene. Base-compositional bias, saturation of substitutions and artificial grouping of the most rapidly evolving lineages are some of the phylogenetic reconstruction artifacts which can also be a reason for topological variation in phylogenetic trees. The fact that previous studies except for Brankovics et al. [23] have used different gene regions may explain why the phylogenies are incongruent. Furthermore, these studies have used only the gene barcoding regions thus reducing the amount of parsimony informative sites to produce a comprehensive evolutionary story. Phylogenies from Brankovics et al. [23] study and the current study shared six whole gene sequences (*tub2*, *cal*, *tef1-α*, *tef3*, *RPB2* and *top1*) and a conserved region of the mitochondrial genome suggesting that the evolutionary history of these combined genes were similar for the isolates in both studies, presenting concordant clades.

The diversity in the dataset could be another reason why the findings from the current study differs from Lombard et al. [19] dataset. They had included strains which cause disease on humans and animals. The genes in these isolates may be subject to different selective pressures in comparison to isolates from agricultural and natural ecosystems. These selective pressures may be inducing different mutation rates in these strains, creating a different evolutionary history and this may be one of the reasons why they had many clades or groups which were absent in the current study and earlier studies.

In summary, Clades 1, 2 and 3 obtained from the three datasets were congruent, suggesting a similar evolutionary pattern for nuclear and mitochondrial genes of the isolates in these clades. There were three isolates: RBG6505, RBG5714 and VPRI11409, which made the other clades incongruent. Isolates RBG6505 and RBG5714 were grouped in Clade 4 of the conserved region of the mitochondrial genome phylogeny but were separate in the whole genome phylogeny. In the nuclear gene phylogeny, RBG 5714 was in Clade 5 and VPRI11409 was in Clade 4.

In the whole genome and nuclear gene phylogenies, Clade 1 had diverged from the other clades early, while the other clades have separated from each other more recently. Clade 1 has been hypothesised as the ancestral clade of FOSC, originating in South East Asia due to the association of Clade 1 ff. sp. with hosts that evolved in that region [16]. This hypothesis was based on the initial study of the species complex. With more sampling of the complex, Clade 1 is found to be an ancestral clade, but does not seem to have originated from South East Asia as it has isolates associated with hosts originated from other regions as well. Clade 1 in the current study contained isolates from natural ecosystems and *F. oxysporum* f.sp. *canariensis* isolates and a single isolate which had been isolated from a symptomatic tomato seedling.

Accurate establishment of species boundaries and delimitation of species is critical to taxonomy and informs biosecurity and disease control. Concatenation analyses of multi-locus DNA sequence data represents a powerful and commonly used approach to understanding independent evolutionary lineages and phylogenetic relationships between isolates. Such data produce well-supported phylogenies which in many instances are inconsistent with the true species tree [35, 55]. Discordance can be masked between individual gene trees if well-supported clades are recognised as distinct species without implementing a careful examination of species boundaries [42]. It is not necessary for every population or lineage in a phylogenetic analysis to be recognised as a species [56].

GCPSR has been previously used in resolving the species complex in *F. oxysporum*. This concept uses discordance between the nodes to find the species boundaries. Discordance arises due to recombination between the genes. Previous studies on resolving the complex identified two ‘species’ [30], three ‘species’ [23] and 21 ‘species’ [19]. Lombard et al. [19] and Laurence et al. [30] used concatenated four and eight barcoding gene regions respectively while Brankovics et al. [23] used concatenated nine genes and the conserved region of the mitochondrial genome. In Laurence et al. [30] GCPSR study, Clade 1 from an earlier study [18] was resolved to being phylogenetic ‘species’ 1 while Clades 2–5 were in phylogenetic ‘species’ 2. In Brankovics et al. [23] study, the three clades were recognised as three ‘species’, while in Lombard et al. [19] study, every lineage from the concatenated multi-locus phylogeny was recognised as a ‘species’.

The current study used a different approach, the MSC model for species delimitation to recognise the species boundaries within the complex. This is a relatively new and arguably successful approach to phylogenomics whereby the evolutionary history of multilocus sequences is explained through gene trees and species trees [57], and gene trees are estimated simultaneously with the species tree for estimating phylogenetic relationships. This model has been successfully used in identification of the number of species in the *Alternaria alternata* [38] and *Colletotrichum* [42] species complexes. As far as we are aware, this is the first time this model has been used for unravelling the species complex in *F. oxysporum.* The model provided very strong support for the three concordant clades from the three datasets in this current study to represent the three ‘species’ within the FOSC (Fig. 4). These species are concordant with the three ‘species’ identified by Brankovics et al. [23] using GCPSR. This concordance between both the datasets having six common genes implies that both models are producing similar results. The model, however, rejected the presence of four, five and seven ‘species’ which were based on the number of clades in conserved mitochondrial genome phylogeny, nuclear gene dataset and whole genome phylogeny and sub-clades of the whole genome phylogeny respectively. Testing the two and three ‘species’ theory of Laurence et al. [30] and Brankovics et al. [23] dataset, using the MSC model, produced high support for the presence of two ‘species’ and three ‘species’ respectively giving the same prediction as that of the GCPSR. Analysis of Lombard et al. [19] dataset resulted in very well supported nodes for only two ‘species’; *F. veterinarium* and *F. oxysporum* (Supplementary Fig. 9). There was also high node support for *Fusarium foetens* which was the outgroup. Their dataset did not have enough information for delimiting the complex into further nineteen species with high node support. A similar finding was also reported by Liu et al. [42] whereby the concatenated multi-locus analysis was not supported by the coalescent based analysis. The potential source of discordance between the two may be explained by incomplete lineage sorting [35] of some genes in some isolates which then delineated as separate lineages. This suggests that species might be overestimated if all well-supported clades from phylogenetic analysis of single or multi-locus DNA sequence on a small sample dataset are accepted as distinct species. The species named as *F. oxysporum* contains the neotype for *F. oxysporum* (*F. oxysporum* isolated from rotten potato tuber from Germany) and correlates to sub-clade in Clade 3 in the current study (Fig. 2 and Supplementary Fig. 7). Clade 3 is represented as ‘species’ 2 in the current study and this may be called *F. oxysporum*. Clades 2 and 3 (‘species’ 3 and 2 respectively) had isolates which were obtained from both the agricultural and natural ecosystems. Clade 1, ‘species’ 1 had only isolates from the natural ecosystems (soil substrate) and *F. oxysporum* f.sp. *canariensis* except for VPRI42181 which was isolated from *Lycopersicon esculentum* (tomato).

## Conclusion

The phylogenetic analyses of the FOSC has demonstrated considerable genetic diversity. Despite this, the current study has shown that there were three clades that were congruent in nearly all the FOSC phylogeny studies. The three datasets used in the current study showed four and five clades within the FOSC but have three clades which were concordant in all the phylogenies. Three ‘species’ were identified within the complex using the MSC model and these three ‘species’ represent the three concordant clades. This result is concordant with Brankovics et al. [23] GCPSR study. Clade 3 which is ‘species’ 2 contains the neotype for *F. oxysporum,* hence clade 3 may be called *F. oxysporum*.

## Methods and materials

### Isolates used

The whole genome was sequenced for a total of 99 isolates obtained from the Victorian Plant Pathogen Herbarium (VPRI) and from the Royal Botanic Gardens (RBG) Sydney collections. Fifty-two of these isolates were obtained from VPRI, with 36 isolates isolated from symptomatic plants sent for diagnosis between 1976 and 2018 (Supplementary Table 2). These isolates were confirmed as *F. oxysporum* using a polymerase chain reaction (PCR)-based assay with RPB1-Fa and RPB1-G2R primers. The amplicons were sequenced and blasted on GenBank and confirmed as *F. oxysporum.*

The remaining 16 isolates were obtained from diseased Canary Island Palm samples sent for diagnosis and were confirmed as *F. oxysporum* f. sp. *canariensis* (Foc) using a PCR-based assay with the Foc-specific primers HK66 + HK67 [58]. Forty-seven isolates were obtained from RBG. Thirteen of these were isolated from natural ecosystem soil (NE) by Laurence et al. [18], two isolates were characterised as *F. oxysporum* f. sp. *niveum* and 32 *Fusarium oxysporum* f.sp. *pisi* (Fop) isolates were collected between 2005 and 2009 during field surveys in Victoria, New South Wales and Queensland from symptomatic *Pisum sativum* (snow pea) plants [59]. Details of these isolates are presented in Supplementary Table 3.

Different sets of reference sequences were used for different phylogenetic analyses. For whole genome phylogenetic analysis, whole genome reference sequences of *F. oxysporum* f. sp. *vasinfectum* (NRRL25433), *F. oxysporum* f. sp. *raphani* (NRRL54005), *F. oxysporum* f. sp. *conglutinans* (NRRL54008), *F. oxysporum* f. sp. *melonis* (NRRL26406), *F. oxysporum* f. sp. *radicis cucumerinum* (Forc016), *F. oxysporum* f. sp. *lycopersici* (Fol4287), two *F. oxysporum* f. sp. *cucumerinum* strains (Foc011, Foc013) and two *F. proliferatum* strains (ITEM2341, NRRL62905) were included from NCBI GenBank.

For nuclear gene phylogenetic analysis, sequences of two *F. oxysporum* f. sp. *cucumerinum* strains (Foc011, Foc016), *F. oxysporum* f. sp. *vasinfectum* (NRRL25433), *F. oxysporum* f. sp. *melonis* (NRRL26406), *F. oxysporum* f. sp. *raphani* (NRRL54005) and *F*. *proliferatum* (ITEM2400) were retrieved from the European Nucleotide Archive (ENA) (https://www.ebi.ac.uk/ena).

For mitochondrial genome analysis, the reference sequences of *F. oxysporum* f. sp. *cucumerinum* (Foc001), *F. oxysporum* f. sp. *cumini* (F11), *F. oxysporum* f. sp. *dianthi* (Fod001), *F. oxysporum* f. sp. *lycopersici* (DF041), *F. oxysporum* f. sp. *niveum* (Fon020), *F. oxysporum* f. sp. *pisi* (NRRL37622), *F. oxysporum* f. sp. *radicis cucumerinum* (Forc016), *F. oxysporum* f. sp. *raphani* (NRRL54005), *F. oxysporum* f. sp. *vasinfectum* (NRRL25433), *F. oxysporum* f. sp. *radicis lycopersici* (NRRL26381) and *F*. *proliferatum* (ITEM2287) were included from ENA (https://www.ebi.ac.uk/ena).

### Culture growth and DNA extraction

All cultures were single spored using the method described by Burgess et al. [60]. Working cultures were maintained on silica gel beads using the procedure described by Leslie et al. [61]. For DNA extraction, cultures were grown on Potato Dextrose Agar (PDA; Diffco Laboratories, Detroit) under dark incubation for 5 days at 25 °C. Two plugs from vigorously growing regions of the culture were cut out and placed in an Eppendorf tube then ground using a sterile micro-pestle before transferring into a Falcon tube containing 45 ml of Potato Dextrose Broth (PDB; Diffco Laboratories, Detroit). These tubes were placed onto a Ratek orbital shaker/mixer and gently shaken at 7RPM in the dark for five days. The resultant mycelia were harvested by filtering each isolate at a time through Miracloth™ (CalBiochem, San Diego, CA) with a pore size of 22–25 μm. This was then washed using sterile water and lyophilised for 48 h. Genomic DNA was extracted using the Cetyl trimethylammonium bromide (CTAB) protocol described by O’Donnell et al. [62].

### Sequencing

Long and short-read sequencing technologies were both used for sequencing the 99 isolates for the current study. For long-read sequencing, MinION was used while for short reads, the Illumina sequencing platform was used.

#### MinION

MinION is a long-read DNA sequencer developed by Oxford Nanopore. Assemblies generated from MinION sequence data were used to guide assembly from the Illumina platform. Eight isolates (two from NE, one *Foc*, five *Fop*) were sequenced on MinION flow cells using the library kit SQK-LSK109 and the protocol as per 1D Genomic DNA by Ligation. Six of these isolates (RBG6477, RBG6423, RBG6462, RBG5783, RBG6313 and VPRI42117) were sequenced on the old flow cell type: FLO-MIN106, while the other 2 isolates (RBG6418, RBG6425) were sequenced on the newly released flow cell version ‘Rev D’ ASIC type: FLO-MIN106D. There were some modifications to the library preparation protocol. The fragmentation step was omitted due to the need for longer reads and the starting material was increased from 1 μg of genomic DNA to2 μg in-order to have at least 1 μg of the finished library for loading onto the FLO-MIN106 flow cell. A single library was loaded per FLO-MIN106 flow cell while for the FLO-MIN106D flow cell, 2 libraries were loaded onto a single flow cell for increased sequencing efficiency.

The raw signal data (fast5 files) from sequencing were basecalled into DNA sequence data in fastq format using Albacore v 2.3.1 (https://nanoporetech.com). Albacore was also used to categorise the basecalled reads based on the average quality score and only reads with average quality score of >Q7 were saved for genome assembly. Adapter sequences were then trimmed from the reads using Porechop v 0.2.1 (https://github.com/rrwick/Porechop) and these reads were saved in fastq format.

#### b) Illumina

Paired-end libraries were prepared for 99 isolates using the Illumina Nextera XT DNA library prep kit according to the manufacturer’s protocols (Illumina). These libraries were sequenced using Illumina HiSeq Rapid. Fastq sequence files generated from the sequencing run were filtered using nuclear software v3.6.16 (GYDLE Inc., Montreal, Canada), filtering sequences based on a minimum length of 50 bp and removal of adaptors. Low-quality reads (<Q20) from the fastq sequence files were filtered using fastp [63].

### Data assembly and analysis

Phylogenetic analyses were carried out using three datasets: mitochondrial genome (conserved region of the mitochondrial genome), whole genome and nuclear gene dataset (concatenated eight nuclear genes). Different procedures were used for preparing and analysing these datasets.

### Mitochondrial genome dataset

#### Mitochondrial genome sequences

The sequence variations in the large variable region (LV) region of the mitochondrial genome has led to three variant mitochondrial genome sequence types: variant 1, 2 and 3. The mitochondrial genome sequences of these variant types include both the conserved region as well as the LV region sequences. Representative sequences of the three variant types were extracted from the ENA. These were LT906338, *F. oxysporum* strain Fon015 (Variant 1), LT906345, *F. oxysporum* strain FOSC3-a (Variant 2) and LT906354, *F. oxysporum* strain NRRL37622 (Variant 3). Fastq reads from the sequencing platforms were first mapped onto these three reference sequences using the program Gydle (GYDLE Inc., Montreal, Canada) to determine the variant type of each isolate. Once the variant types of the isolates were determined, then the fastq reads for each isolate were mapped to the respective variant type reference sequence to determine the mitochondrial genome sequence by resolving for SNPs and indels using Gydle (GYDLE Inc., Montreal, Canada) (Sawbridge, pers. comm.). One Way ANOVA and Fisher’s Test for Least Significant Difference were tested for the mitochondrial genome length, length of the conserved region and LV region using the statistical software OriginPro 2019 (www.originlab.com) (Supplementary Figs. S2a, S2b and S2c) and box and whisker plots were prepared in Excel to show the mean, the quartiles and the range (Supplementary Fig. S3).

#### Phylogenetic analysis

Mitochondrial genomes were aligned as described by Brankovics et al. [23]. The mitochondrial consensus sequences were annotated using MFannot (http://megasun.bch.umontreal.ca/cgi-bin/mfannot/mfannotInterface.pl). All the variant type genome sequences were split into two regions: the large variable region (between the *trnT*(*tgt*) and the *nad2* gene) and the remaining part of the genome, which is referred to as the conserved part of the mitochondrial genome. The sequences for both regions were divided into non-overlapping homologous blocks of 7000 bp. These 7000 bp blocks were aligned using ClustalW [60] in BioEdit [61] and concatenated in the order of extraction. The most appropriate substitution model was determined for the conserved and LV regions of the mitochondrial genomes using MEGA X [62].

A Maximum likelihood (ML) tree was generated with 1000 bootstrap replications using MEGA X [62] with *F. proliferatum* (ITEM2287) as an outgroup. The tree was condensed with bootstrap node support cut off value of 70%. Bayesian Inference (BI) analysis was performed with MRBAYES 3.2.6 [63] plugin in Geneious R11.1.5 (http://www.geneious.com) using 4,000,000 generations of MCMC and a burn-in of 10,000 trees. A consensus tree was constructed from raw trees with the support threshold set at 95%. *F. proliferatum* (ITEM2287) was used as an outgroup.

#### Comparison to earlier studies

Brankovics et al. [23] dataset consisted of sixty-one European plant pathogenic strains (i.e. a number of *formae speciales*), isolates from soil and Fo47 (biological control strain). Ten of the mitochondrial genome sequences of the three variant mitochondrial genome types identified by Brankovics et al. [23] and representing all the three clades in their study were used as references. Sequences of eight Variant 1 type isolates: *F. oxysporum* f.sp. *cucumerinum* (Foc001- LT906307) (clade 1), *F. oxysporum* f.sp. *cumini* (F11-LT841205) (clade 3), *F. oxysporum* f.sp. *dianthi* (Fod001-LT841219) (clade 2), *F. oxysporum* f.sp. *niveum* (Fon020-LT906340) (clade 2), *F. oxysporum* f.sp. *radicis*-*cucumerinum* (Forc016-LT906342) (clade 3), *F. oxysporum* f.sp. *radicis-lycopersici* (NRRL26381-LT906352) (clade 3), *F. oxysporum* f.sp. *raphani* (NRRL54005-LT906356) (clade 2) and *F. oxysporum* f.sp. *vasinfectum* (NRRL25433-LT906351) (clade 2), one Variant 2 isolate: *F. oxysporum* f.sp. *lycopersici* (DF041-LT906304) (clade 3) and one Variant 3 isolate: *F. oxysporum* f.sp. *pisi* (NRRL37622-LT906354) (clade 2) were extracted from ENA and included as reference sequences with the mitochondrial genome dataset from the current study. A ML tree was generated as described earlier. Since majority of the isolates in the current study had variant 1 type mitochondrial genome, more variant 1 type reference isolates were included in the current study.

### Whole genome dataset

#### Genome sequences

Two different programs were used as de novo assemblers for the isolates used in this study. For the eight isolates that were sequenced on MinION, reads from MinION sequencing were used for de novo assembly using Canu [64]. These assemblies were visualised on Bandage [65] and contigs with <5X coverage and length < 200 bp were removed. The assembled genomes then went through many rounds of polishing with Illumina reads using Unicycler [66]. The reads of the remaining isolates that were sequenced only using Illumina were de novo assembled using SPAdes [67]. Once again, contigs <5X coverage and < 200 bp were removed.

To measure the quality and completeness of the assembled genome, a quantitative assessment of the assembly was carried out based on evolutionary informed expectations of gene content known as Benchmarking Universal Single-Copy Orthologs (BUSCO) [68] (https://busco.ezlab.org/). Expectation of single-copy genes to be found in the genome are evolutionarily sound [69]. BUSCO identifies complete, duplicated, fragmented and missing genes through comparison of different datasets. *F. oxysporum* is a filamentous ascomycetous fungus belonging to the order Nectriaceae, family Hypocreales and class Sordariomycetes. The assembled genomes were compared using BUSCO to the class Sordariomycetes as previously described by Armitage et al. [70]. Assemblies having greater than 90% of 3725 core Sordariomycete genes as estimated by BUSCO were kept for further analysis.

Data generated from the MinION sequencing platform were assembled in far fewer contigs [37–45, 48–67] compared to those generated on Illumina (892–12,866). MinION assemblies were more accurate and complete (in terms of number of genes present) with BUSCO scores > 98% while Illumina assemblies ranged from 90 to 99% (data not shown). Both the short and long reads sequencing technologies were combined to get the good attributes from both platform (Illumina accurate reads, MinION long reads).

#### Phylogenetic analysis

Ten whole genome sequences (different *formae speciales*) were extracted from NCBI: *F. oxysporum* f.sp. *cucumerinum* (Foc011- MABT00000000.1, Foc013-MABJ00000000.1), *F. oxysporum* f.sp. *melonis* (NRRL26406- NJCY00000000.1), *F. oxysporum* f.sp. *lycopersici*(Fol4287-QESU00000000.1), *F. oxysporum* f.sp. *radicis-cucumerinum* (Forc016-MABQ00000000.2), *F. oxysporum* f.sp. *conglutinans* (NRRL54008- AGNF00000000.1), *F. oxysporum* f.sp. *raphani* (NRRL54005-AGNG00000000.1), *F. oxysporum* f.sp. *vasinfectum* (NRRL25433-AGNC00000000.1) and *F. proliferatum* (ITEM2341-PKMI00000000.1 and NRRL62905-FCQG00000000.1) and were combined with the whole genome dataset of the current study. All these sequences were annotated using AUGUSTUS [71]. Nucleotide sequences for the isolates were added to the respective GFF annotated files. These files were the input files for Roary: the pan-genome pipeline [72, 73]. Using BLASTP (NCBI) and TRIBE-Markov cluster (MCL) algorithm [45], it clusters the proteins into orthologs (removes any paralogs from the core genes). The protein sequences of the orthologous groups are concatenated and aligned using Multiple Alignment FAST Fourier Transform (MAFFT) [46]. A ML tree was generated using FastTree [47] with the General Time Reversal (GTR) substitution model. FastTree uses the Shimodaira-Hasegawa (SH) test for three alternate topologies for every split and each split is sampled 1000 times. *F. proliferatum* (ITEM2341 and NRRL62905) were used as an outgroup.

### Nuclear gene dataset

#### Nuclear gene sequences

Reference sequences for eight single copy protein coding genes: β-tubulin II (*tub2*) (LT905861) from *F. oxysporum* f.sp. *melonis*, calmodulin (*cal*) (LT905875) from *F. oxysporum* f.sp. *melonis*, the largest and second largest subunit of DNA-dependent RNA polymerase II (*RPB1* (KX434921)and *RPB2* (LT841210), from *F. oxysporum* and *F. oxysporum* f.sp. *cumini* respectively), translation elongation factor 1α (*tef1-α*) (LT841203) from *F. oxysporum* f.sp. *cumini*, translation elongation factor 3 (*tef3*) (LT905812) from *F. oxysporum* f.sp. *lycopersici*, topoisomerase I (*top1*) (LT905754) from *F. oxysporum* f.sp. *cumini* and *mitochondrial small-subunit ribosomal RNA (mtSSU) (EU313488) from F. oxysporum* f.sp. *pisi* were extracted from ENA and NCBI. Using the program BLASTDB (NCBI), these reference sequences were mapped to the whole genome, and the mapped region was extracted as the respective gene sequences for the isolates. Five isolates (Foc011, Forc016, NRRL25433, NRRL26406 and NRRL54005) representing the three clades from Brankovics et al. [23] study were added as reference isolates. Since *RPB1* and *mtSSU* sequences were not in Brankovics et al. [23] dataset, sequences for these genes were extracted and added to the sequences of *tub2*, *cal*, *RPB2*, *tef1-α*, *tef3* and *top1* in their dataset. These are the six genes that are common between their study and the current study. *RPB1* gene sequences for Foc011 (LT905647), Forc016 (LT906089), NRRL25433 (LT906206), NRRL26406 (LT906232) and NRRL54005 (LT906271) were extracted from ENA and added to the other six genes. Sequences for the *mtSSU* for these isolates were not in any database hence it had to be extracted from the mitochondrial genome sequences of these isolates available on ENA (Foc011-LT906308, Forc016-LT906342, NRRL25433-LT906351, NRRL26406-LT906353 and NRRL54005- LT906356). These were the only isolates from which *mtSSU* sequences could be extracted from their mitochondrial genome using the program BLASTDB (NCBI) with *mtSSU* sequence from *F. oxysporum* f.sp. *pisi* (EU313488) as a reference sequence. The protein coding gene sequences of *tub2*-LT841257, *cal*-LT841258, *RPB2*-LT841266, *tef1-α-LT841259 tef3*-LT841260 *top1*, *RPB1*-LT841265 for *F. proliferatum* (ITEM24000) were extracted from ENA. Sequence for the *mtSSU* was extracted from the mitochondrial genome (LT841261) using the program BLASTDB (NCBI) with *mtSSU* sequence from *F. oxysporum* f.sp. *pisi* (EU313488) as a reference sequence. *F. proliferatum* was used as an outgroup

#### Phylogenetic analyses

Multiple sequence alignments for each of the eight genes were carried out using ClustalW [74] in BioEdit [75]. The sequence alignments for individual loci were viewed using BioEdit Sequence Alignment Editor [75] and columns containing gaps were removed. Phylogenetic trees were constructed for each single locus as well as a concatenated nuclear gene dataset. The most appropriate substitution model was determined for each of the single locus and nuclear gene datasets using MEGA X [76]. ML trees for single loci and the combined dataset were generated using MEGA X [76] with 1000 bootstrap replications. For the combined dataset, the tree was condensed with bootstrap node support cut off value of 70%. Bayesian inference (BI) analysis was carried out on single loci and combined datasets using the MRBAYES 3.2.6 [77] plugin in Geneious R11.1.5 (http://www.geneious.com) with 4,000,000 generations of Markov chain Monte Carlo (MCMC) and a burn-in of 10,000 trees. The combined dataset was partitioned to reflect the most appropriate nucleotide substitution model for each single locus. A consensus tree was constructed from raw trees with the support threshold set at 95%. *F. proliferatum* ITEM2400 was used as an outgroup.

#### Comparison to earlier studies

##### Lombard’s dataset

The Lombard et al. [19] dataset consisted of four barcoding gene sequences (*cal*, *RPB2*, *tef1-α* and *tub2*) from isolates from the culture collection of the Westerdijk Fungal Biodiversity Institute and included human, animal and plant pathogenic strains as well as isolates from soil. The gene sequences for 52 isolates representing the 21 ‘species’ and the outgroup from their study were added to the sequences from the current study. These gene sequences were concatenated into a combined dataset. Alignment and phylogenetic tree generation were carried out as described earlier except that ML trees generated were not condensed.

##### Brankovics’s dataset

Brankovics et al. [23] dataset consisted of sequences from 10 loci (*act* (γ-actin), *tub2*, *cal*, *tef1-α, tef3, rpl10a* (60S ribosomal protein L10), *RPB2, top1,* rDNA repeat and the conserved region of the mitochondrial genome). They used European plant pathogenic strains, isolates from soil and they had also included Fo47 (strain that is used as a biological control). Since *act*, *rpl10a* and *rDNA* repeat gene sequences were not part of the current dataset, the sequences for these genes were extracted from the whole genome sequences of all the isolates using BLASTDB (NCBI) and concatenated with the respective gene sequences in Brankovics et al. [23] dataset. The best nucleotide substitution model was selected and a ML phylogenetic tree was constructed using MEGA [76].

##### Laurence’s dataset

Laurence et al. [18] studied the diversity of *F. oxysporum* from Australian natural ecosystems, grouping them into five clades using barcoding gene sequences of *tef1-α* and *mtSSU* [18], later dividing them into 2 phylogenetic species [30] using GCPSR with eight barcoding gene sequences (*mtSSU*, *RPB1*, *RPB2*, *cal*, *tef1-α, nir* (nitrate reductase)*, PHO,* (Phosphate permease) and *acl1* (ATP citrate lyase). All Clade 1 isolates were phylogenetic ‘species’ 1, while phylogenetic species 2 had isolates from the remaining clades. Fourteen isolates from their study representing the five clades were included with the dataset from the current study. *PHO*, *nir* and *acl1* gene sequences were omitted from the current study dataset, so reference sequences for *nir* (EU246653), *PHO* (EU246663), *acl1* (LT841199) were extracted from ENA. Using the program BLASTDB (NCBI), these reference sequences were mapped to the whole genome, and the mapped region was extracted as the respective gene sequences for the isolates. All the gene sequences from current study were concatenated with respective gene sequences from Laurence et al. [18] study. Alignment and phylogenetic tree generation were carried out as mentioned earlier except that ML trees generated were not condensed.

##### O’Donnell’s dataset

O’Donnell et al. [16] pioneered the use of phylogenetics to study the relationship between members of the species complex, identifying three clades in their dataset. They used barcoding gene sequences of *tef1-α* and *mtSSU* to find phylogenetic relationships between American plant pathogenic strains and isolates from soil. The two gene regions for three isolates per Clade 1 (NRRL26024, NRRL26029, NRRL26035), Clade 2 (NRRL25356, NRRL25367, NRRL25607) and Clade 3 (NRRL22555, NRRL26037, NRRL26406) were extracted from NCBI. These gene sequences were added to the respective gene sequences of the isolates in the current study. These gene sequences were concatenated into a combined dataset. Alignment and phylogenetic tree generation were carried out as described earlier except that ML trees generated were not condensed.

#### Multi species coalescent (MSC) model-based species tree estimation-*BEAST

The MSC model describes the stochastic process of lineage joining when one traces the genealogical history of a gene sequence from a population backward in time until their most recent common ancestor. MSC was applied to the eight single copy nuclear genes (*tub2*, *cal*, *RPB1*, *RPB2*, *tef1-α*, *tef3*, *top1* and *mtSSU*). These genes were concatenated for phylogenetic analysis but for species delimitation, these genes were not concatenated but used as single loci for construction of single gene trees. The MSC model was not applied to the mitochondrial genome dataset because it reflects the history of the maternal lineages, which are often incongruent with the species’ history [78]. Additionally, the MSC model was not used with the whole genome dataset as this dataset consists of 6800 genes and to prepare single gene sequences of 6800 genes for 99 isolates was not practical.

The *fasta* file alignments for individual genes were converted to a Nexus format file in Geneious R11.1.5 (http://www.geneious.com) and imported into the *BEAST template in BEAUti2 [79] for the generation of XML format files. For the needed prior in *BEAST, the isolates were assigned to potential species (to be tested) under the Taxon sets tab. Isolates falling in different clades in different phylogenetic analyses were tested by assigning the respective species name under the Taxon sets tab. For example, isolates that were in clade 1 were assigned as species 1. The nucleotide substitution model for *tef1-α* and *tef3* was TN93 while HKY was chosen for the other genes. The clock model was set as relaxed clock log-normal and species tree prior was set as the Yule model. The datasets were run three times with 2 billion MCMC generations, sampling every 5000 generations. Tracer 1.7 [80] was used to view the log file generated by BEAST2 [79] to ensure equilibrium had been reached. The maximum clade credibility tree constructed from all the gene trees was generated with posterior probability of each node computed after a 15% burn-in using TreeAnnotator v1.6.1 [81] and this was then viewed on FigTree v1.4.2 [82]. Proposals for different number of species within the FOSC were tested based on the number of well supported clades in different phylogenies. Three (number of concordant clades from all the phylogenies), four (number of clades in the conserved mitochondrial genome phylogeny), five (number of clades in the whole genome and nuclear gene dataset phylogenies) and seven (number of sub-clades in the whole genome dataset) were tested as the potential number of species.

#### Comparison to earlier studies

Previous species delimitation studies in FOSC identified different number of putative species: two species from Laurence et al. [30] study, three species from Brankovics et al. [23] study and 21 species from Lombard et al. [19] study. All the isolates were assigned to the respective species numbers as per these studies and analysed using MSC model as described previously.

## Supplementary information

**Additional file 1: Supplementary Table 1.** Mitochondrial genome lengths, Variant types and intron present in the Fusarium oxysporum isolates used in this study. ^1^corrected mt length refers to the length without the introns present.

**Additional file 2: Supplementary Table 2.** Details of the hosts and symptoms from which the VPRI *Fusarium oxysporum* isolates were obtained.

**Additional file 3: Supplementary Table 3.** Details of the isolates used in the current study. ^1^ RBG: Royal Botanic Gardens, Sydney collection, Australia; VPRI: Victorian Plant Pathogen Herbarium, Australia. ^2^ VPRI10351 and VPRI11409 are not Australian isolates.

**Additional file 4: Supplementary Figure 1.** One Way ANOVA and Fisher test for Least Significant Difference between the length of the large variable region of different variant types present in the *Fusarium oxysporum* isolates used for phylogenetic analyses

**Additional file 5: Supplementary Figure 2.** Box and whisker plots showing the spread of Fusarium oxysporum mitochondrial genome length, length of the conserved region and large variable region.

**Additional file 6: Supplementary Figure 3.** One Way ANOVA and Fisher test for Least Significant Difference between the mitochondrial genome length of different variant types present in the *Fusarium oxysporum* isolates used for phylogenetic analyses

**Additional file 7: Supplementary Figure 4.** One Way ANOVA and Fisher test for Least Significant Difference between the length of the conserved region of different variant types present in the *Fusarium oxysporum* isolates used for phylogenetic analyses

**Additional file 8: Supplementary Figure 5.** Maximum likelihood consensus tree with bootstrap node support of > 70% was inferred from the sequences of the large variable region of Variant 1 *Fusarium oxysporum* isolates. Eight reference isolates (Foc001, NRRL25433, Fon002, Fod001, NRRL54005, F11, NRRL26381 and Forc016) from Brankovics et al. [23] were included in the analysis. The tree was rooted to *Fusarium proliferatum* (ITEM2287).

**Additional file 9: Supplementary Figure 6.** Maximum likelihood consensus tree with bootstrap node support of > 70% was inferred from the sequences of the large variable region of Variant 2 and 3 *Fusarium oxysporum* isolates. One reference isolate per variant type (V2-DF041, V3-NRRL37622) from Brankovics et al. [23] was included in the analysis. The trees were not rooted.

**Additional file 10: Supplementary Figure 7.** Maximum likelihood tree generated from the combined dataset of Lombard et al. [19] (52 isolates) and isolates from the current study using MEGA X with 1000 bootstrap replications. The analysis was based on concatenated partial gene sequences of *cal*, *RPB2*, *tef1-α* and *tub2*. Isolates representing the neotype of *F. oxysporum* are labelled and highlighted in red. Isolates from the current study have the prefixes RBG and VPRI before the numbers. Isolates included from Lombard et al. [19] dataset have prefixes NRRL and CBS before the numbers. The tree was rooted to *Fusarium udum* (CBS177.31) and *Fusarium foetens* (CBS120665).

**Additional file 11: Supplementary Figure 8.** Maximum likelihood tree generated from the combined dataset of Laurence et al. [30] (14 isolates) and isolates from the current study using MEGA X with 1000 bootstrap replications. The analysis was based on concatenated partial gene sequences of *mtSSU*, *RPB1*, *RPB2*, *cal*, *tef1-α, nir, PHO,* and *acl1*. The tree was rooted to *Fusarium proliferatum* (ITEM2400). Isolates from the current study have the prefixes RBG and VPRI before the numbers while isolates with prefix AUST belong to Laurence et al. [30]. The isolates belonging to Clade 1 (phylogenetic species 1) are highlighted in orange and this is concordant with Clade 1 and species 1 in the current study.

**Additional file 12: Supplementary Figure 9.** Species tree estimation of Lombard et al. [19] multi-locus DNA sequence dataset using multispecies coalescent (MSC) model in *BEAST. The species denoted by 21, 17 and 9 are F*. veterinarium*, *F. oxysporum* and *F. foetens* respectively. *Fusarium foetens* is the outgroup. Numbers above branches indicate node support as posterior probabilities.

## Data Availability

The assembled sequences used in the analysis of the current study has been deposited into the National Center for Biotechnology Information (NCBI) with the following accession numbers:MN451075-MN451173, MN457051-MN457149, MN457150-MN457248, MN456952-MN457050, MN457249-MN457347, MN457348-MN457446, MN457546-MN457644, MN457546-MN457644, MN511185-MN511283 and WGOF00000000.1-WGRZ00000000.1.

## Abbreviations

*acl1*: ATP citrate lyase
*atp6*: Mitochondrially encoded ATP synthase membrane subunit 6 gene
BI: Bayesian inference
BLASTP: Basic Local Alignment Search Tool-Protein
*cal*: Calmodulin
*CAN*: *F. oxysporum* f. sp. c*anariensis*
*cob*: Mitochondrially encoded cytochrome b
ENA: European Nucleotide Archive
f.sp.: *Forma specialis*
ff. spp.: *Formae speciales*
*Foc*: *F. oxysporum* f.sp. *canariensis*
*Fop*: *Fusarium oxysporum* f.sp. *pis*i
FOSC: *Fusarium oxysporum* species complex
GCPSR: Genealogical Concordance Phylogenetic Species Recognition
*IEL*: Independent evolutionary lineages
LV: Large variable region
MCL: Markov Cluster
MCMC: Markov chain Monte Carlo
ML: Maximum likelihood
MSC: Multispecies coalescent
mt: Mitochondrial
*mtSSU rDNA*: Mitochondrial small subunit
*nad2*: Mitochondrially encoded NADH dehydrogenase 2
*nad5*: Mitochondrially encoded NADH dehydrogenase 5
NCBI: National Center for Biotechnology Information
NE: Natural ecosystems
*NIR*: Nitrate reductase
PDA: Potato dextrose agar
PDB: Potato dextrose broth
*PHO*: Phosphate permease
RGB: Royal Botanic Gardens
*rnl*: Mitochondrial LSU rRNA gene
*RPB1*: Largest subunit of DNA-dependent RNA polymerase II
*RPB2*: Second largest subunit of DNA-dependent RNA polymerase II
*tef-1α*: Translation elongation factor
*Top1*: Topoisomerase I
*tub2*: β-tubulin II
VPRI: Victorian Plant Pathology Herbarium

## References

[1] Dean R, Van Kan JA, Pretorius ZA, Hammond-Kosack KE, Di Pietro A, Spanu PD (2012). The top 10 fungal pathogens in molecular plant pathology. Mol Plant Pathol.

[2] Baayen RP (2000). Diagnosis and detection of host-specific forms of *Fusarium oxysporum*. EPPO Bulletin.

[3] Lievens B, Rep M, Thomma BP (2008). Recent developments in the molecular discrimination of *formae speciales* of *Fusarium oxysporum*. Pest Manag Sci.

[4] Kuldau GA, Yates IE, Bacon CW, Jr White JF (2000). Evidence for *Fusarium* endophytes in cultivated and wild plants. Microbial Endophytes.

[5] Fravel D, Olivain C, Alabouvette C (2003). *Fusarium oxysporum* and its biocontrol. New Phytol.

[6] Bao J, Fravel D, Lazarovits G, Chellemi D, van Berkum P, O’Neill N (2004). Biocontrol genotypes of *Fusarium oxysporum* from tomato fields in Florida. Phytoparasitica.

[7] Fracchia S, Garcia-Romera I, Godeas A, Ocampo J (2000). Effect of the saprophytic fungus *Fusarium oxysporum* on arbuscular mycorrhizal colonization and growth of plants in greenhouse and field trials. Plant Soil.

[8] O'Donnell K, Sutton DA, Rinaldi MG, Magnon KC, Cox PA, Revankar SG (2004). Genetic diversity of human pathogenic members of the *Fusarium oxysporum* complex inferred from multilocus DNA sequence data and amplified fragment length polymorphism analyses: evidence for the recent dispersion of a geographically widespread clonal lineage and nosocomial origin. J Clin Microbiol.

[9] O’Donnell K, Sutton D, Wiederhold N, Robert V, Crous P, Geiser D. Veterinary Fusarioses within the United States. J Clin Microbiol. 2016;5(11):2813–9. 10.1128/JCM.01607-16 PMC5078561 27605713

[10] Snyder WC, Hansen HN (1940). The species concept in *Fusarium*. Am J Bot.

[11] Kistler H. Genetic diversity in the plant-pathogenic fungus *Fusarium oxysporum*. Phytopathology. 1997;87(4):474–9. 10.1094/PHYTO.1997.87.4.474. 10.1094/PHYTO.1997.87.4.474 18945129

[12] Snyder WC, Hansen H. Variation and speciation in the genus *Fusarium*. Ann N Y Acad Sci. 1954;60(1):16–23. 10.1111/j.1749-6632.1954.tb39994.x. 10.1111/j.1749-6632.1954.tb39994.x 13229216

[13] Demers JE, Gugino BK, Jimenez-Gasco M (2015). Highly diverse endophytic and soil *Fusarium oxysporum* populations associated with field-grown tomato plants. Appl Environ Microbiol.

[14] Edel-Hermann V, Lecomte C (2019). Current status of *Fusarium oxysporum formae speciales* and races. Phytopathology.

[15] Katan T, Primo PD (1999). Current status of vegetative compatibility groups in *Fusarium oxysporum*. Phytoparasitica.

[16] O'Donnell K, Kistler HC, Cigelnik E, Ploetz RC (1998). Multiple evolutionary origins of the fungus causing Panama disease of banana: concordant evidence from nuclear and mitochondrial gene genealogies. Proc Natl Acad Sci U S A.

[17] Baayen RP, O'Donnell K, Bonants PJ, Cigelnik E, Kroon LP, Roebroeck EJ (2000). Gene genealogies and AFLP analyses in the *Fusarium oxysporum* complex identify monophyletic and nonmonophyletic *formae speciales* causing wilt and rot disease. Phytopathology.

[18] Laurence MH, Burgess LW, Summerell BA, Liew ECY (2012). High levels of diversity in *Fusarium oxysporum* from non-cultivated ecosystems in Australia. Fungal Biol.

[19] Lombard L, Sandoval-Denis M, Lamprecht SC, Crous PW (2019). Epitypification of *Fusarium oxysporum*-clearing the taxonomic chaos. Persoonia.

[20] Burger G, Gray MW, Lang FB (2003). Mitochondrial genomes: anything goes. Trends Genet.

[21] Lang FB, Gray MW, Burger B (1999). Mitochondrial genome evolution and the origin of eukaryotes. Annu Rev Genet.

[22] Al-Reedy RM, Malireddy R, Dillman CB, Kennell JC (2012). Comparative analysis of *Fusarium* mitochondrial genomes reveals a highly variable region that encodes an exceptionally large open reading frame. Fungal Genet Biol.

[23] Brankovics B, van Dam P, Rep M, de Hoog GS, van der Lee TA J, Waalwijk C (2017). Mitochondrial genomes reveal recombination in the presumed asexual *Fusarium oxysporum* species complex. BMC Genomics.

[24] Fourie G, van Der Merwe N, Wingfield B, Bogale M, Tudzynski B, Wingfield M, et al. Evidence for inter-specific recombination among the mitochondrial genomes of *Fusarium* species in the *Gibberella fujikuroi* complex. BMC Genomics. 2013;14(1):4770–81. 10.1186/1471-2164-14-605. 10.1186/1471-2164-14-605 PMC3847072 24010864

[25] Skovgaard K, Nirenberg HI, O'Donnell K, Rosendahl S (2001). Evolution of *Fusarium oxysporum* f. sp *vasinfectum* races inferred from multigene genealogies. Phytopathology.

[26] O'Donnell K, Gherbawy Y, Schweigkofler W, Adler A, Prillinger H (1999). Phylogenetic analyses of DNA sequence and RAPD data compared in *Fusarium oxysporum* and related species from maize. J Phytopathol.

[27] van Dam P, Fokkens L, Ayukawa Y, van der Gragt M, ter Horst A, Brankovics B (2017). A mobile pathogenicity chromosome in *Fusarium oxysporum* for infection of multiple cucurbit species. Sci Rep.

[28] Fourie G, Steenkamp ET, Gordon TR, Viljoen A (2009). Evolutionary relationships among the *Fusarium oxysporum* f. sp. *cubense* vegetative compatibility groups. Appl Environ Microbiol.

[29] Yang Z, Rannala B (2010). Bayesian species delimitation using multilocus sequence data. Proc Natl Acad Sci U S A.

[30] Laurence MH, Summerell BA, Burgess LW, Liew ECY (2014). Genealogical concordance phylogenetic species recognition in the *Fusarium oxysporum* species complex. Fungal Biol.

[31] Dettman JR, Jacobson DJ, Taylor JW (2003). A multilocus genealogical approach to phylogenetic species recognition in the model eukaryote Neurospora. Evolution.

[32] Taylor JW, Jacobson DJ, Kroken S, Kasuga T, Geiser DM, Hibbett DS (2000). Phylogenetic species recognition and species concepts in fungi. Fungal Genet Biol.

[33] Avise JC, Ball JRM (1990). Principles of genealogical concordance in species concepts and biological taxonomy. Oxf Surv Evol Biol.

[34] Degnan J, Rosenberg N (2009). Gene tree discordance, phylogenetic inference and the multispecies coalescent. Trends Ecol Evol.

[35] Kubatko LS, Degnan JH (2007). Inconsistency of phylogenetic estimates from concatenated data under coalescence. Syst Biol.

[36] Heled J, Drummond AJ (2010). Bayesian inference of species trees from multilocus data. Mol Biol Evol.

[37] Rannala B, Yang Z (2003). Bayes estimation of species divergence times and ancestral population sizes using DNA sequences from multiple loci. Genetics.

[38] Stewart JE, Timmer LW, Lawrence CB, Pryor BM, Peever TL (2014). Discord between morphological and phylogenetic species boundaries: incomplete lineage sorting and recombination results in fuzzy species boundaries in an asexual fungal pathogen. BMC Evol Biol.

[39] Waters JM, Rowe DL, Burridge CP, Wallis GP (2010). Gene trees versus species trees: reassessing life-history evolution in a freshwater fish radiation. Syst Biol.

[40] Satler JD, Carstens BC, Hedin M (2013). Multilocus species delimitation in a complex of morphologically conserved trapdoor spiders (*Mygalomorphae, Antrodiaetidae*, *Aliatypus*). Syst Biol.

[41] Cranston KA, Hurwitz B, Ware D, Stein L, Wing RA (2009). Species trees from highly incongruent gene trees in rice. Syst Biol.

[42] Liu F, Wang M, Damm U, Crous PW, Cai L. Species boundaries in plant pathogenic fungi: a *Colletotrichum* case study. BMC Evol Biol. 2016;16(81). 10.1186/s12862-016-0649-5. 10.1186/s12862-016-0649-5 PMC4832473 27080690

[43] Li W, Godzik A (2006). Cd-hit: a fast program for clustering and comparing large sets of protein or nucleotide sequences. Bioinformatics.

[44] Huang Y, Niu B, Gao Y, Fu L, Li W (2010). CD-HIT suite: a web server for clustering and comparing biological sequences. Bioinformatics.

[45] Enright AJ, Van Dongen S, Ouzounis CA (2002). An efficient algorithm for large-scale detection of protein families. Nucleic Acids Res.

[46] Katoh K, Misawa K, Kuma K, Miyata T. MAFFT: a novel method for rapid multiple sequence alignment based on fast fourier transform. Nucleic Acids Res. 2002;30. 10.1093/nar/gkf436. 10.1093/nar/gkf436 PMC135756 12136088

[47] Price MN, Dehal PS, Arkin AP (2009). FastTree: computing large minimum-evolution trees with profiles instead of a distance matrix. Mol Biol Evol.

[48] van Dam P, de Sain M, Ter Horst A, van der Gragt M, Rep M (2018). Comparative genomics-based markers: discrimination of host-specificity in *Fusarium oxysporum*. Appl Environ Microbiol.

[49] Snyder WC, Hansen HN (1945). The species concept in *Fusarium* with reference to discolor and other sections. Am J Bot.

[50] van Dam P, Fokkens L, Schmidt S, Linmans J, Kistler H, Ma L, et al. Effector profiles distinguish *formae speciales* of *Fusarium oxysporum*. Environ Microbiol. 2016;18(11):4087–102. 10.1111/1462-2920.13445. 10.1111/1462-2920.13445 27387256

[51] Xu J, Li H (2015). Current perspectives on mitochondrial inheritance in fungi. Cell Health Cytoskeleton.

[52] Ploetz RPK. Fusarium wilt of banana and Wallace's line: Was the disease originally restricted to his Indo-Malayan region? Australas Plant Pathol Soc News. 1997;26(239–249). 10.1071/AP97039.

[53] Ma LJ, van der Does HC, Borkovich KA, Coleman JJ, Daboussi MJ, Di Pietro A (2010). Comparative genomics reveals mobile pathogenicity chromosomes in *Fusarium*. Nature.

[54] Castresana J (2007). Topological variation in single-gene phylogenetic trees. Genome Biol.

[55] Degnan J, Rosenberg N (2006). Discordance of species trees with their most likely gene trees. PLoS Genet.

[56] Sukumaran J, Knowles L (2017). Multispecies coalescent delimits structure, not species. Proc Natl Acad Sci U S A.

[57] Liu L, Yu L, Kubatko L, Pearl DK, Edwards SV. Coalescent methods for estimating phylogenetic trees. Mol Phylogenet Evol. 2009;53(1):320–328 doi: https://doi.org/10.1016/j.ympev.2009.05.033. 10.1016/j.ympev.2009.05.033 19501178

[58] Mercier S, Louvet J (1973). Recherches sur les fusarioses. X. Une fusariose vasculaire (*Fusarium oxysporum*) du palmier des Canaries (*Phoenix canariensis*). Ann Phytopathol.

[59] Watson A, Yousiph A, Liew E, Duff J. Fusarium wilt of snow peas. Industry and Investment NSW and Horticulture Australia Limited in partnership with AUSVEG; 2009.

[60] Burgess LW, Summerell BA, Bullock S, Gott KP, Backhouse D. Laboratory manual for Fusarium research. 3rd ed. Sydney: University of Sydney; 1994.

[61] Leslie JF, Summerell BA (2006). The *Fusarium* laboratory manual.

[62] O'Donnell K, Cigelnik E (1997). Two divergent intragenomic rDNA ITS2 types within a monophyletic lineage of the fungus *Fusarium* are nonorthologous. Mol Phylogenet Evol.

[63] Chen S, Zhou Y, Chen Y, Gu J (2018). Fastp: an ultra-fast all-in-one FASTQ preprocessor. Bioinformatics.

[64] Koren S, Walenz BP, Berlin K, Miller JR, Bergman NH, Phillippy AM (2017). Canu: scalable and accurate long-read assembly via adaptive k-mer weighting and repeat separation. Genome Res.

[65] Wick RR, Schultz MB, Zobel J, Holt KE (2015). Bandage: interactive visualisation of de novo genome assemblies. Bioinformatics.

[66] Wick RR, Judd LM, Gorrie CL, Holt KE (2017). Unicycler: resolving bacterial genome assemblies from short and long sequencing reads. PLoS Comput Biol.

[67] Bankevich A, Nurk S, Antipov D, Gurevich AA, Dvorkin M, Kulikov AS (2012). SPAdes: a new genome assembly algorithm and its applications to single-cell sequencing. J Comput Biol.

[68] Waterhouse RM, Seppey M, Simão FA, Manni M, Ioannidis P, Klioutchnikov G (2017). BUSCO applications from quality assessments to gene prediction and phylogenomics. Mol Biol Evol.

[69] Simão FA, Waterhouse RM, Ioannidis P, Kriventseva EV, Zdobnov EM (2015). BUSCO: assessing genome assembly and annotation completeness with single-copy orthologs. Bionformatics.

[70] Armitage AD, Taylor A, Sobczyk MK, Laura B, L, Greenfield BPJ, Bates HJ, et al. Characterisation of pathogen-specific regions and novel effector candidates in *Fusarium oxysporum* f. sp. *cepae*. Sci Rep 2018;8(1):1–15 doi: 10.1038/s41598-018-30335-7. 10.1038/s41598-018-30335-7 PMC6131394 30202022

[71] Stanke M, Morgenstern B (2005). AUGUSTUS: a web server for gene prediction in eukaryotes that allows user-defined constraints. Nucleic Acids Res.

[72] Seemann T (2014). Prokka: rapid prokaryotic genome annotation. Bioinformatics.

[73] Page AJ, Cummins CA, Hunt M, Wong VK, Reuter S, Holden MT (2015). Roary: rapid large-scale prokaryote pan genome analysis. Bioinformatics.

[74] Thompson JD, Higgins DG, Gibson TJ (1994). CLUSTAL W: improving the sensitivity of progressive multiple sequence alignment through sequence weighting, position-specific gap penalties and weight matrix choice. Nucleic Acids Res.

[75] Hall T (1999). BioEdit: a user-friendly biological sequence alignment editor and analysis program for windows 95/98/NT. Nucleic Acids Symp Ser.

[76] Kumar S, Stecher G, Li M, Knyaz C, Tamura K (2018). MEGA X: Molecular evolutionary genetics analysis across computing platforms. Mol Biol Evol.

[77] Huelsenbeck J, Ronquist F (2001). MRBAYES: Bayesian inference of phylogenetic trees. Bioinformatics.

[78] Moreyra NN, Mensch J, Hurtado J, Almeida F, Laprida C, Hasson E (2019). What does mitogenomics tell us about the evolutionary history of the *Drosophila buzzatii* cluster (repleta group)?. PloS One.

[79] Bouckaert R, Vaughan TG, Barido-Sottani J, Duchêne S, Fourment M, Gavryushkina A (2019). BEAST 2.5: An advanced software platform for Bayesian evolutionary analysis. PLoS Comput Biol.

[80] Rambaut A, Drummond AJ, Xie D, Baele G, Suchard MA (2018). Posterior summarization in bayesian phylogenetics using tracer 1.7. Syst Biol.

[81] Rambaut A, Drummond A. TreeAnnotator version 1.6.1. 2010.

[82] Rambaut A. FigTree v1.4.2. 2014.

