# Supplementary material for: Phylogenetic relationship between Australian Fusarium oxysporum isolates and resolving the species complex using the multispecies coalescent model
Source: BMC Genomics. 2020 Mar 20;21:248. doi: 10.1186/s12864-020-6640-y (PMC7085163; doi:10.1186/s12864-020-6640-y)
Supplement: Supplementary file 1 — Additional file 1: Supplementary Table 1. Mitochondrial genome lengths, Variant types and intron present in the Fusarium oxysporum isolates used in this study. 1corrected mt length refers to the length without the introns present. [file 12864_2020_6640_MOESM1_ESM.pdf]

strain	Species	*forma specialis*	Clade	Introns present in gene			mt length (bp)	corrected mt length (bp)[1]	conserved region length (bp)	LV region length (bp)	LV variant type
				atp6	cob(1)	cob(2)					
RBG6358	*F. oxysporum*		1	-	-	-	43295	42286	33533	9762	1
VPRI42420	*F. oxysporum*	*canariensis*	1	+	+	+	50427	45710	33630	16797	1
VPRI41208	*F. oxysporum*	*canariensis*	1	-	+	+	48677	45199	33432	15245	1
VPRI32288	*F. oxysporum*	*canariensis*	1	-	-	-	39441	38431	28267	11174	1
VPRI32441	*F. oxysporum*	*canariensis*	1	-	+	-	43469	42038	33332	10137	1
VPRI41207	*F. oxysporum*	*canariensis*	1	-	+	-	42999	41632	31583	11416	1
VPRI32287	*F. oxysporum*	*canariensis*	1	+	-	-	43356	41787	32721	10635	1
VPRI42181	*F. oxysporum*		1	-	+	-	46943	44696	33563	13380	1
RBG5689	*F. oxysporum*		1	-	-	-	45388	44378	33574	11814	1
RBG5831	*F. oxysporum*		1	-	+	+	44169	40690	26976	17193	2
RBG5844	*F. oxysporum*		1	-	-	-	38116	37106	32307	5809	3
RBG5862	*F. oxysporum*		1	+	-	-	46556	45217	33496	13060	1
RBG6301	*F. oxysporum*		1	-	-	-	42199	41189	30678	11521	1
RBG6313	*F. oxysporum*		1	-	+	-	43295	42037	33565	9730	1
RBG6309	*F. oxysporum*		1	-	-	-	43825	42816	34505	9320	1
VPRI42889	*F. oxysporum*		2	-	-	-	50019	49009	33393	16626	2
VPRI10358	*F. oxysporum*		2	-	-	-	50208	49918	33296	16912	2
VPRI42760	*F. oxysporum*		2	-	-	-	45795	44785	33406	12389	1
VPRI42339	*F. oxysporum*	*canariensis*	2	-	-	-	45922	44912	33401	12521	1
VPRI32442	*F. oxysporum*	*canariensis*	2	-	-	-	46061	45051	33401	12660	1
VPRI43193	*F. oxysporum*	*canariensis*	2	-	-	-	45843	44833	33401	12442	1
VPRI42119	*F. oxysporum*	*canariensis*	2	-	-	-	33510	32500	22680	10830	1
VPRI42327	*F. oxysporum*	*canariensis*	2	-	-	-	45061	44052	33842	11219	1
VPRI42253	*F. oxysporum*		2	-	-	-	45700	44690	33195	12505	1
VPRI42198	*F. oxysporum*		2	-	-	-	45929	44919	33421	12508	1
VPRI42190	*F. oxysporum*		2	-	-	-	45769	44759	33318	12451	1
VPRI41836	*F. oxysporum*		2	-	-	-	45848	44838	33415	12433	1
VPRI41778	*F. oxysporum*		2	-	-	-	46009	44999	33422	12587	1
VPRI19293	*F. oxysporum*		2	-	-	-	45894	44884	33438	12456	1
VPRI17796	*F. oxysporum*		2	-	-	-	46018	45008	33412	12606	1
VPRI16963	*F. oxysporum*		2	-	-	-	45963	44953	33424	12539	1
VPRI13039	*F. oxysporum*		2	-	-	-	45521	44511	33401	12120	1
VPRI11762	*F. oxysporum*		2	-	-	-	45698	44688	33398	12300	1
VPRI10403	*F. oxysporum*		2	-	-	-	43510	42500	33424	10086	1
RBG5783	*F. oxysporum*		2	-	-	-	45981	44975	33432	12549	1
RBG6400	*F. oxysporum*	*pisi*	2	-	-	-	43503	42493	32563	10940	1
RBG6418	*F. oxysporum*	*pisi*	2	-	-	-	45688	44678	33424	12264	1
RBG6423	*F. oxysporum*	*pisi*	2	-	-	-	42075	41065	32445	9630	1
RBG6433	*F. oxysporum*	*pisi*	2	-	-	-	44760	43750	33020	11740	1
RBG6454	*F. oxysporum*	*pisi*	2	-	-	-	44515	43505	32365	12150	1
RBG6475	*F. oxysporum*	*pisi*	2	-	-	-	44639	43629	32153	12486	1
RBG6503	*F. oxysporum*	*pisi*	2	-	-	-	42328	41322	32017	10311	1
VPRI11235	*F. oxysporum*		2	-	-	-	45668	44658	33401	12267	1
VPRI42109	*F. oxysporum*		2	-	-	-	42850	41840	33407	9443	1
VPRI42888	*F. oxysporum*		2	-	-	-	45920	44910	33606	12314	1

VPRI42180	*F. oxysporum*		2	-	-	-	43338	42329	34453	8885	1
VPRI42252	*F. oxysporum*		2	-	-	-	43146	42137	32661	10485	1
RBG6324	*F. oxysporum*		2	-	-	-	45711	44702	34432	11279	1
RBG6396	*F. oxysporum*	*pisi*	2	-	-	-	42509	41500	33817	8692	1
RBG6397	*F. oxysporum*	*pisi*	2	-	-	-	45192	44183	34174	11018	1
RBG6398	*F. oxysporum*	*pisi*	2	-	-	-	45039	44030	33790	11249	1
RBG6406	*F. oxysporum*	*pisi*	2	-	-	-	44775	43766	34419	10356	1
RBG6416	*F. oxysporum*	*pisi*	2	-	-	-	45708	44699	34448	11260	1
RBG6417	*F. oxysporum*	*pisi*	2	-	-	-	41359	40350	31303	10056	1
RBG6419	*F. oxysporum*	*pisi*	2	-	-	-	42632	41623	33766	8866	1
RBG6420	*F. oxysporum*	*pisi*	2	-	-	-	45704	44695	34432	11272	1
RBG6421	*F. oxysporum*	*pisi*	2	-	-	-	44803	43794	33559	11244	1
RBG6422	*F. oxysporum*	*pisi*	2	-	-	-	42934	41925	32513	10421	1
RBG6425	*F. oxysporum*	*pisi*	2	-	-	-	41485	40476	32831	8654	1
RBG6431	*F. oxysporum*	*pisi*	2	-	-	-	43398	42389	32634	10764	1
RBG6442	*F. oxysporum*	*pisi*	2	-	-	-	45714	44705	34430	11284	1
RBG6448	*F. oxysporum*	*pisi*	2	-	-	-	44535	43526	33271	11264	1
RBG6450	*F. oxysporum*	*pisi*	2	-	-	-	44558	43549	34433	10125	1
RBG6494	*F. oxysporum*	*pisi*	2	-	-	-	45495	44486	34399	11096	1
RBG6499	*F. oxysporum*	*pisi*	2	-	-	-	42884	41875	34399	8485	1
RBG7064	*F. oxysporum*	*niveum*	2	-	-	-	50071	49062	33810	16261	2
RBG7070	*F. oxysporum*	*niveum*	2	-	-	-	44854	43845	34427	10427	1
VPRI11409	*F. oxysporum*		3	-	+	-	46302	44055	33054	13248	1
VPRI32264	*F. oxysporum*		3	-	+	-	52595	50348	33623	18972	2
VPRI16234	*F. oxysporum*		3	-	+	-	52604	50357	33512	19092	2
VPRI11681	*F. oxysporum*		3	-	+	-	52623	50376	33512	19111	2
VPRI42118	*F. oxysporum*	*canariensis*	3	-	+	-	47470	45223	33289	14181	1
VPRI42117	*F. oxysporum*	*canariensis*	3	-	-	-	45074	44064	34176	10898	1
VPRI43195	*F. oxysporum*	*canariensis*	3	-	+	-	46304	43050	36473	9831	1
VPRI43194	*F. oxysporum*	*canariensis*	3	-	-	-	44048	43038	31289	12759	1
VPRI31638	*F. oxysporum*		3	-	+	-	47439	45190	33531	13908	1
VPRI17577	*F. oxysporum*		3	-	+	-	47519	45272	33333	14186	1
VPRI12300	*F. oxysporum*		3	-	+	-	47289	45042	33289	14000	1
VPRI10605	*F. oxysporum*		3	-	+	-	44389	42142	33606	10783	1
RBG5833	*F. oxysporum*		3	-	-	-	45811	44801	33091	12720	1
RBG5836	*F. oxysporum*		3	-	-	-	33040	32030	27738	5302	3
RBG6462	*F. oxysporum*	*pisi*	3	-	+	-	37255	35940	27660	9595	1
RBG6464	*F. oxysporum*	*pisi*	3	-	-	-	42375	41365	31663	10712	1
RBG6477	*F. oxysporum*	*pisi*	3	-	+	-	46818	45380	33599	13219	1
RBG6480	*F. oxysporum*	*pisi*	3	-	-	-	42217	41207	29376	12841	1
RBG6429	*F. oxysporum*	*pisi*	3	-	-	-	45653	44644	34606	11047	1
RBG6444	*F. oxysporum*	*pisi*	3	-	-	-	44884	43875	34456	10428	1
RBG6466	*F. oxysporum*	*pisi*	3	-	+	-	43781	42280	33581	10200	1
RBG6505	*F. oxysporum*	*pisi*	4	-	-	-	45758	44749	34467	11291	1
VPRI10351	*F. oxysporum*	*tracheiphilum*	3	-	-	-	43185	42175	30620	12565	1
VPRI10405	*F. oxysporum*		3	-	-	-	44112	44112	32438	11674	1
VPRI10408	*F. oxysporum*		3	-	-	-	45533	44523	33197	12336	1
VPRI16235	*F. oxysporum*		3	-	-	-	38478	37468	31811	6667	3
VPRI32289	*F. oxysporum*		3	-	-	-	45473	44464	34146	11327	1
VPRI41884	*F. oxysporum*		3	-	+	-	39428	37952	33510	5918	3

VPRI41920	*F. oxysporum*		3	-	-	-	33236	32226	22431	10805	1
VPRI42176	*F. oxysporum*		3	-	-	-	40624	39614	30253	10371	1
VPRI42882	*F. oxysporum*		3	-	+	-	45472	44055	33781	11691	1
RBG5714	*F. oxysporum*		4	-	-	-	36676	35670	30046	6630	3
