# Supplementary material for: Phylogenetic relationship between Australian Fusarium oxysporum isolates and resolving the species complex using the multispecies coalescent model
Source: BMC Genomics. 2020 Mar 20;21:248. doi: 10.1186/s12864-020-6640-y (PMC7085163; doi:10.1186/s12864-020-6640-y)
Supplement: Supplementary file 2 — Additional file 2: Supplementary Table 2. Details of the hosts and symptoms from which the VPRI Fusarium oxysporum isolates were obtained. [file 12864_2020_6640_MOESM2_ESM.pdf]

Supplementary Table 2. Details of host and symptoms from which the VPRI *Fusarium oxysporum* isolates were obtained

Accession	Host/substrate	Year of collection	Location	Symptoms on the host
VPRI32441	*Phoenix canariensis*	2002	Geelong, VIC	Palm dieback (pathogenic)
VPRI32442	*Phoenix canariensis*	2002	Geelong, VIC	Palm dieback (pathogenic)
VPRI42420	*Phoenix canariensis*	2014	South Yarra, VIC	Palm dieback (pathogenic)
VPRI41208	*Phoenix canariensis*	2005	South Yarra, VIC	Palm dieback (pathogenic)
VPRI41207	*Phoenix canariensis*	2005	South Yarra, VIC	Palm dieback (pathogenic)
VPRI32287	*Phoenix canariensis*	2005	South Yarra, VIC	Palm dieback (pathogenic)
VPRI32288	*Phoenix canariensis*	2005	South Yarra, VIC	Palm dieback (pathogenic)
VPRI42117	soil associated with *Phoenix canariensis*	2012	Bendigo, VIC	Palm dieback (pathogenic)
VPRI42119	soil associated with *Phoenix canariensis*	2012	Bendigo, VIC	Palm dieback (pathogenic)
VPRI42118	soil associated with *Phoenix canariensis*	2012	Bendigo, VIC	Palm dieback (pathogenic)
VPRI42327	*Phoenix canariensis*	2013	Port Melbourne, VIC	Palm dieback (pathogenic)
VPRI42339	*Phoenix canariensis*	2014	St. Kilda, VIC	Palm dieback (pathogenic)
VPRI43193	*Phoenix canariensis*	2017	Middle Park, VIC	Palm dieback (pathogenic)
VPRI43194	*Phoenix canariensis*	2017	Melbourne, VIC	Palm dieback (pathogenic)
VPRI43195	*Phoenix canariensis*	2017	St. Kilda, VIC	Palm dieback (pathogenic)
VPRI32289	*Phoenix canariensis*	2005	South Yarra, VIC	Palm dieback (pathogenic)
VPRI41778	*Aloe* x *spinosissima*	2010	South Yarra, VIC	Leaf rot (may be pathogenic)
VPRI42888	*Malus pumila*	2017	Shepparton, VIC	Associated with graft union failure (may not be pathogenic)
VPRI41836	*Phaseolus vulgaris*	2009	Lindenow, VIC	Plant wilt (pathogenic)
VPRI42889	*Phaseolus vulgaris*	2017	Wodonga, VIC	Plant stunting and russeting caused by insects, no typical Fusarium wilting symptoms. (may not be pathogenic)
VPRI11235	*Begonia* sp.	1981	Bendigo, VIC	Collar rot; associated with *F. subglutinans*. (May not be pathogenic)
VPRI13039	*Capsicum annum*	1985	Werribee South, VIC	Yellowing of leaves and wilting of plants. (pathogenic)

VPRI10358	*Dianthus caryophyllus*	1976	Burnley, VIC	Stem rot (pathogenic)
VPRI11762	*Dianthus caryophyllus*	1982	Portsea, VIC	Stem rot; associated with *Rhizoctonia solani* (pathogenic)
VPRI19293	*Dianthus caryophyllus*	1990	Dingley, VIC	Stem rot (pathogenic)
VPRI42252	*Pinus* sp.	2013	Silvan,VIC	Death of tree. Associated with *F. solani* which was determined to be the disease-causing agent. (may not be pathogenic)
VPRI42253	*Pinus* sp.	2013	Silvan,VIC	Death of tree. Associated with *F. solani* which was determined to be the disease-causing agent. (may not be pathogenic)
VPRI41920	*Encephalartos ferox*	2011	South Yarra, VIC	Leaf rot. Associated with *Phytophthora cinnamomi* which was determined to be the disease-causing agent. (may not be pathogenic)
VPRI17577	*Linum usitatissimum*	1991	Hamilton, VIC	Seedling wilt (pathogenic)
VPRI10605	*Hyacinthus orientalis*	1978	Burnley, VIC	Flower rot; associated with *F. redolens*. (may not be pathogenic)
VPRI31638	*Lupin albus*	2003	Rutherglen, VIC	Reported as pathogen
VPRI42760	*Zea mays*	2016	Newmerella, VIC	Isolated from leaves associated with bacterial cause (may not be pathogenic)
VPRI10408	*Solanum tuberosum*	1962	Koo Wee Rup, VIC	Reported as pathogen
VPRI12300	*Solanum tuberosum*	1984	Thorpdale, VIC	Tuber rot (pathogenic)
VPRI16234	*Solanum tuberosum*	1989	Otway, VIC	Tuber rot (pathogenic)
VPRI16235	*Solanum tuberosum*	1989	Lake Bolac, VIC	Tuber rot (pathogenic)
VPRI16963	*Solanum tuberosum*	1990	Tynong North, VIC	Tuber rot (pathogenic)
VPRI42198	*Solanum tuberosum*	2013	Tasmania	Stem end discoloration (pathogenic)
VPRI17796	*Atriplex* sp.	1992	Tatura, VIC	Stem rot (pathogenic)
VPRI41884	*Nassella trichotoma*	2011	Bathurst, NSW	Grass dieback but not the causal agent (may not be pathogenic)
VPRI42109	*Tibouchina sp.*	2012	Wonga Park, VIC	Associated with *F. sterilihyphosum* (may not be pathogenic)
VPRI11681	*Lycopersicon esculentum*	1982	Frankston, VIC	Plant wilt (pathogenic)
VPRI32264	*Lycopersicon esculentum*	2005	Echuca, VIC	Plant wilt (pathogenic)
VPRI42180	*Lycopersicon esculentum*	2013	Queenscliff, VIC	Seedling wilt- no internal discoloration (may be pathogenic)
VPRI42181	*Lycopersicon esculentum*	2013	Queenscliff, VIC	Seedling wilt- discoloration in crown and lower stem (pathogenic)

VPRI42190	soil associated with *Lycopersicon esculentum*	2013	Queenscliff, VIC	Death of several plants in the field but no symptoms were recorded. Was associated with other pathogenic fungi (may be pathogenic)
VPRI42882	*Juglans regia*	2017	Leeton, NSW	Isolated from fruit (may not be pathogenic)
VPRI10403	*Triticum aestivum*	1970	Balliang, VIC	Reported as pathogen
VPRI10405	*Triticum aestivum*	1971	Burramine, VIC	Root rot (pathogenic)
