# Supplementary material for: Phylogenetic relationship between Australian Fusarium oxysporum isolates and resolving the species complex using the multispecies coalescent model
Source: BMC Genomics. 2020 Mar 20;21:248. doi: 10.1186/s12864-020-6640-y (PMC7085163; doi:10.1186/s12864-020-6640-y)
Supplement: Supplementary file 3 — Additional file 3: Supplementary Table 3. Details of the isolates used in the current study. 1 RBG: Royal Botanic Gardens, Sydney collection, Australia; VPRI: Victorian Plant Pathogen Herbarium, Australia. 2 VPRI10351 and VPRI11409 are not Australian isolates. [file 12864_2020_6640_MOESM3_ESM.pdf]

Supplementary Table 3. Details of the Isolates used in the current study.

Accession[1]	*Formae speciales*	Host/substrate	Year of collection	Location
RBG5831	*F. oxysporum*	soil	2012	Wilsons Promontory,VIC
RBG5844	*F. oxysporum*	soil	2012	Mount Kaputar, NSW
RBG5862	*F. oxysporum*	soil	2012	Great Sandy, QLD
RBG5836	*F. oxysporum*	soil	2012	Grampians, VIC
RBG5833	*F. oxysporum*	soil	2012	Grampians, VIC
RBG6301	*F. oxysporum*	soil	2012	Mount Kaputar, NSW
RBG6309	*F. oxysporum*	soil	2012	Carnarvon Gorge, QLD
RBG6313	*F. oxysporum*	soil	2012	Gibraltar Range, NSW
RBG6324	*F. oxysporum*	soil	2012	Werrikimbe, NSW
RBG6358	*F. oxysporum*	soil	2012	Carnarvon Gorge, QLD
RBG5689	*F. oxysporum*	soil	2012	Deep Water, QLD
RBG5714	*F. oxysporum*	soil	2012	Kalbarri, WA
RBG5783	*F. oxysporum*	soil	2012	Kimberly, WA
RBG6480	*pisi*	*Pisum sativum*	2003-2009	Sydney, NSW
RBG6416	*pisi*	*Pisum sativum*		Bairnsdale, VIC
RBG6466	*pisi*	*Pisum sativum*		Sydney, NSW
RBG6462	*pisi*	*Pisum sativum*		Sydney, NSW
RBG6420	*pisi*	*Pisum sativum*		Bairnsdale, VIC
RBG6419	*pisi*	*Pisum sativum*		Bairnsdale, VIC
RBG6398	*pisi*	*Pisum sativum*		Korumburra, VIC
RBG6417	*pisi*	*Pisum sativum*		Bairnsdale, VIC
RBG6442	*pisi*	*Pisum sativum*		Bairnsdale, VIC
RBG6400	*pisi*	*Pisum sativum*		Korumburra, VIC
RBG6422	*pisi*	*Pisum sativum*		Bairnsdale, VIC
RBG6477	*pisi*	*Pisum sativum*		Sydney, NSW
RBG6475	*pisi*	*Pisum sativum*		Sydney, NSW
RBG6464	*pisi*	*Pisum sativum*		Sydney, NSW
RBG6494	*pisi*	*Pisum sativum*		Bundaberg, QLD
RBG6423	*pisi*	*Pisum sativum*		Bairnsdale, VIC
RBG6433	*pisi*	*Pisum sativum*		Bairnsdale, VIC
RBG6431	*pisi*	*Pisum sativum*		Bairnsdale, VIC
RBG6421	*pisi*	*Pisum sativum*		Bairnsdale, VIC
RBG6505	*pisi*	*Pisum sativum*		Gympie, QLD
RBG6444	*pisi*	*Pisum sativum*		Bairnsdale, VIC
RBG6450	*pisi*	*Pisum sativum*		Melbourne, VIC
RBG6454	*pisi*	*Pisum sativum*		Sydney, NSW
RBG6397	*pisi*	*Pisum sativum*		Korumburra, VIC
RBG6425	*pisi*	*Pisum sativum*		Bairnsdale, VIC
RBG6503	*pisi*	*Pisum sativum*		Bundaberg, QLD
RBG6499	*pisi*	*Pisum sativum*		Bundaberg, QLD
RBG6429	*pisi*	*Pisum sativum*		Bairnsdale, VIC
RBG6448	*pisi*	*Pisum sativum*		Melbourne, VIC
RBG6396	*pisi*	*Pisum sativum*		Korumburra, VIC
RBG6406	*pisi*	*Pisum sativum*		Bairnsdale, VIC
RBG6418	*pisi*	*Pisum sativum*		Bairnsdale, VIC
RBG7064	*niveum*	*Citrullus lanatus*	2017	NSW

RBG7070	*niveum*	*Citrullus lanatus*	2017	NSW
VPRI32441	*canariensis*	*Phoenix canariensis*	2002	Geelong, VIC
VPRI32442	*canariensis*	*Phoenix canariensis*	2002	Geelong, VIC
VPRI42420	*canariensis*	*Phoenix canariensis*	2014	South Yarra, VIC
VPRI41208	*canariensis*	*Phoenix canariensis*	2005	South Yarra, VIC
VPRI41207	*canariensis*	*Phoenix canariensis*	2005	South Yarra, VIC
VPRI32287	*canariensis*	*Phoenix canariensis*	2005	South Yarra, VIC
VPRI32288	*canariensis*	*Phoenix canariensis*	2005	South Yarra, VIC
VPRI42117	*canariensis*	soil associated with *Phoenix canariensis*	2012	Bendigo, VIC
VPRI42119	*canariensis*	soil associated with *Phoenix canariensis*	2012	Bendigo, VIC
VPRI42118	*canariensis*	soil associated with *Phoenix canariensis*	2012	Bendigo, VIC
VPRI42327	*canariensis*	*Phoenix canariensis*	2013	Port Melbourne, VIC
VPRI42339	*canariensis*	*Phoenix canariensis*	2014	St. Kilda, VIC
VPRI43193	*canariensis*	*Phoenix canariensis*	2017	Middle Park, VIC
VPRI43194	*canariensis*	*Phoenix canariensis*	2017	Melbourne, VIC
VPRI43195	*canariensis*	*Phoenix canariensis*	2017	St. Kilda, VIC
VPRI32289	*canariensis*	*Phoenix canariensis*	2005	South Yarra, VIC
VPRI41778	*F. oxysporum*	Aloe x spinosissima	2010	South Yarra, VIC
VPRI42888	*F. oxysporum*	*Malus pumila*	2017	Shepparton, VIC
VPRI41836	*F. oxysporum*	*Phaseolus vulgaris*	2009	Lindenow, VIC
VPRI42889	*F. oxysporum*	*Phaseolus vulgaris*	2017	Wodonga, VIC
VPRI11235	*F. oxysporum*	*Begonia* sp.	1981	Bendigo, VIC
VPRI13039	*F. oxysporum*	*Capsicum annum*	1985	Werribee South, VIC
VPRI10358	*F. oxysporum*	*Dianthus caryophyllus*	1976	Burnley, VIC
VPRI11762	*F. oxysporum*	*Dianthus caryophyllus*	1982	Portsea, VIC
VPRI19293	*F. oxysporum*	*Dianthus caryophyllus*	1990	Dingley, VIC
VPRI42252	*F. oxysporum*	*Pinus* sp.	2013	Silvan,VIC
VPRI42253	*F. oxysporum*	*Pinus* sp.	2013	Silvan,VIC
VPRI41920	*F. oxysporum*	*Encephalartos ferox*	2011	South Yarra, VIC
VPRI17577	*F. oxysporum*	*Linum usitatissimum*	1991	Hamilton, VIC
VPRI10351 [2]	*F. oxysporum*	*Glycine max*	1976	USSR
VPRI10605	*F. oxysporum*	*Hyacinthus orientalis*	1978	Burnley, VIC
VPRI31638	*F. oxysporum*	*Lupin albus*	2003	Rutherglen, VIC
VPRI42760	*F. oxysporum*	*Zea mays*	2016	Newmerella, VIC
VPRI10408	*F. oxysporum*	*Solanum tuberosum*	1962	Koo Wee Rup, VIC
VPRI12300	*F. oxysporum*	*Solanum tuberosum*	1984	Thorpdale, VIC
VPRI16234	*F. oxysporum*	*Solanum tuberosum*	1989	Otway, VIC
VPRI16235	*F. oxysporum*	*Solanum tuberosum*	1989	Lake Bolac, VIC
VPRI16963	*F. oxysporum*	*Solanum tuberosum*	1990	Tynong North, VIC
VPRI42198	*F. oxysporum*	*Solanum tuberosum*	2013	Tasmania
VPRI17796	*F. oxysporum*	*Atriplex* sp.	1992	Tatura, VIC
VPRI41884	*F. oxysporum*	*Nassella trichotoma*	2011	Bathurst, NSW
VPRI42109	*F. oxysporum*	*Tibouchina* sp.	2012	Wonga Park, VIC
VPRI11681	*F. oxysporum*	*Lycopersicon esculentum*	1982	Frankston, VIC
VPRI32264	*F. oxysporum*	*Lycopersicon esculentum*	2005	Echuca, VIC
VPRI42180	*F. oxysporum*	*Lycopersicon esculentum*	2013	Queenscliff, VIC
VPRI42181	*F. oxysporum*	*Lycopersicon esculentum*	2013	Queenscliff, VIC

VPRI42190	*F. oxysporum*	soil associated with *Lycopersicon esculentum*	2013	Queenscliff, VIC
VPRI11409 [2]	*F. oxysporum*		1981	unknown
VPRI42882	*F. oxysporum*	*Juglans regia*	2017	Leeton, NSW
VPRI10403	*F. oxysporum*	*Triticum aestivum*	1970	Balliang, VIC
VPRI10405	*F. oxysporum*	*Triticum aestivum*	1971	Burramine, VIC
VPRI42176	*F. oxysporum*	*Schlumbergera truncata*	2012	Narrewarren North, VIC
