# Supplementary material for: Phylogenetic relationship between Australian Fusarium oxysporum isolates and resolving the species complex using the multispecies coalescent model
Source: BMC Genomics. 2020 Mar 20;21:248. doi: 10.1186/s12864-020-6640-y (PMC7085163; doi:10.1186/s12864-020-6640-y)

Supplementary Fig. 1

ANOVAOneWay (23/07/2019 09:35:5

One Way ANOVA

Overall ANOVA

	DF	Sum of Squares	Mean Square	F Value	Prob>F
Model	2	4.17459E8	2.0873E8	108.42369	1.20356E-26
Error	109	2.09839E8	1925129.06796		
Total	111	6.27298E8			

Null Hypothesis: The means of all levels are equal.
Alternative Hypothesis: The means of one or more levels are different.
At the 0.05 level, the population means are significantly different.

Fit Statistics

	R-Square	Coeff Var	Root MSE	Data Mean
	0.66549	0.11906	1387.4902	11653.28571

Means Comparisons

Fisher Test

	MeanDiff	SEM	t Value	Prob	Alpha	Sig	LCL	UCL
V2 V1	6231.39286	542.46634	11.48715	1.71428E-20	0.05	1	5156.24221	7306.54351
V3 V1	-5441.55	635.82789	-8.55821	8.10594E-14	0.05	1	-6701.7402	-4181.3598
V3 V2	-11672.94286	812.43108	-14.36792	6.62601E-27	0.05	1	-13283.15484	-10062.73088

Sig equals 1 indicates that the difference of the means is significant at the 0.05 level.
Sig equals 0 indicates that the difference of the means is not significant at the 0.05 level.

Means Plot (SD as Error)

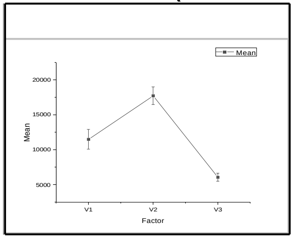

Supplement: Supplementary file 4 — Additional file 4: Supplementary Figure 1. One Way ANOVA and Fisher test for Least Significant Difference between the length of the large variable region of different variant types present in the Fusarium oxysporum isolates used for phylogenetic analyses [file 12864_2020_6640_MOESM4_ESM.pdf]
