# Supplementary figures and images for: Phylogenetic relationship between Australian Fusarium oxysporum isolates and resolving the species complex using the multispecies coalescent model
Source: BMC Genomics. 2020 Mar 20;21:248. doi: 10.1186/s12864-020-6640-y (PMC7085163; doi:10.1186/s12864-020-6640-y)

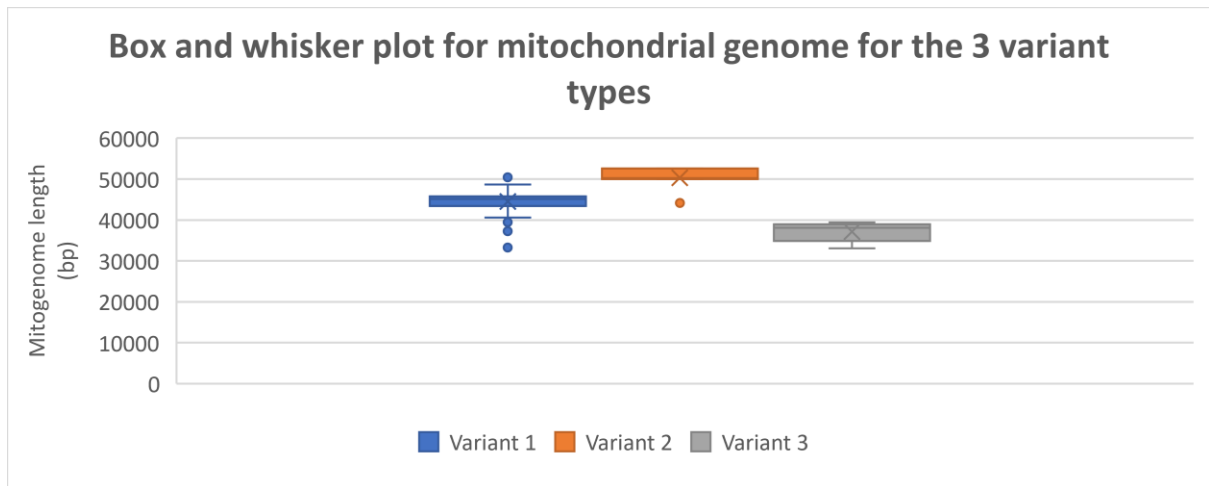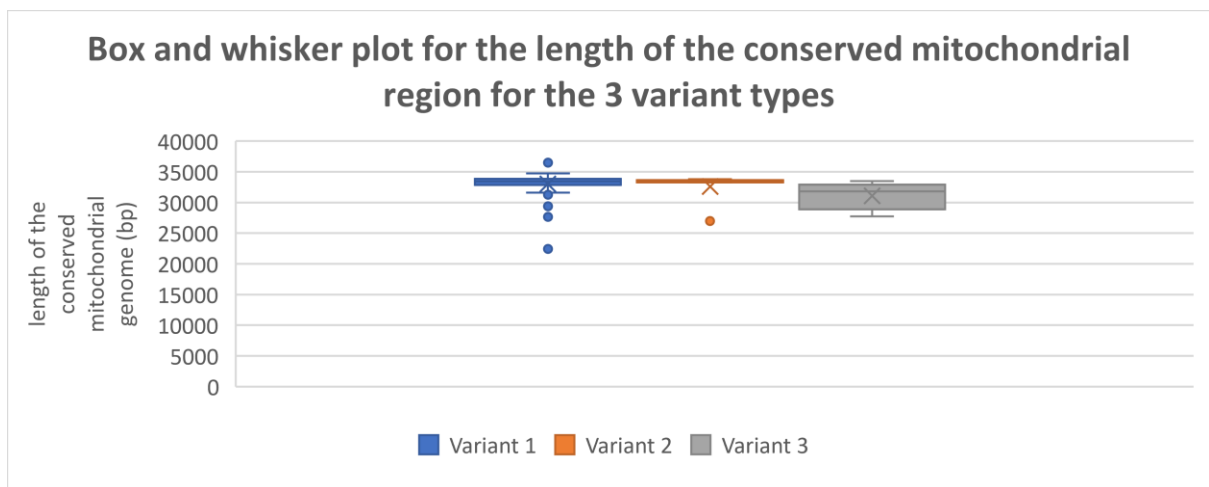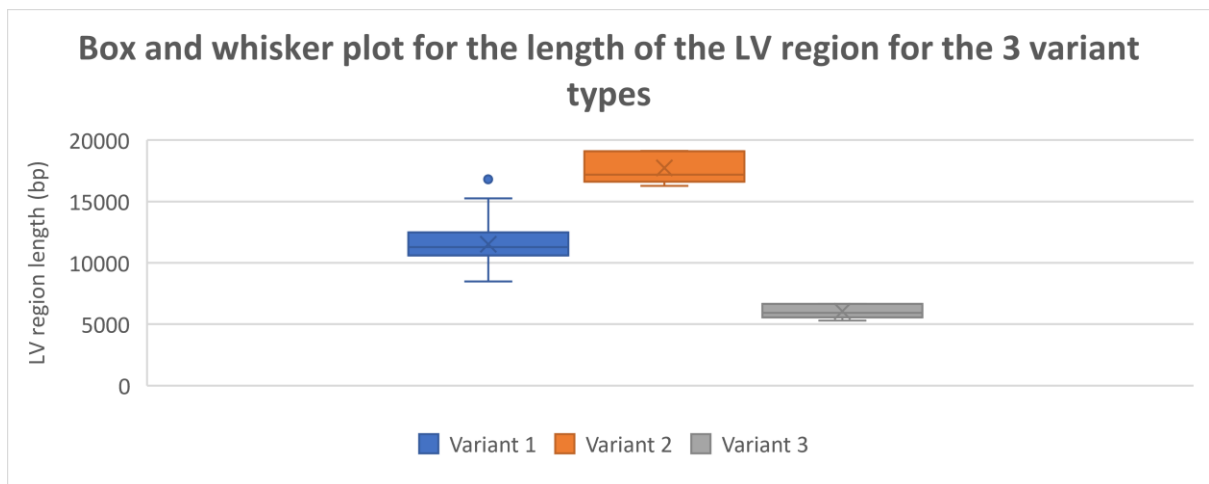

Supplement: Supplementary file 5 — Additional file 5: Supplementary Figure 2. Box and whisker plots showing the spread of Fusarium oxysporum mitochondrial genome length, length of the conserved region and large variable region. [file 12864_2020_6640_MOESM5_ESM.pdf]

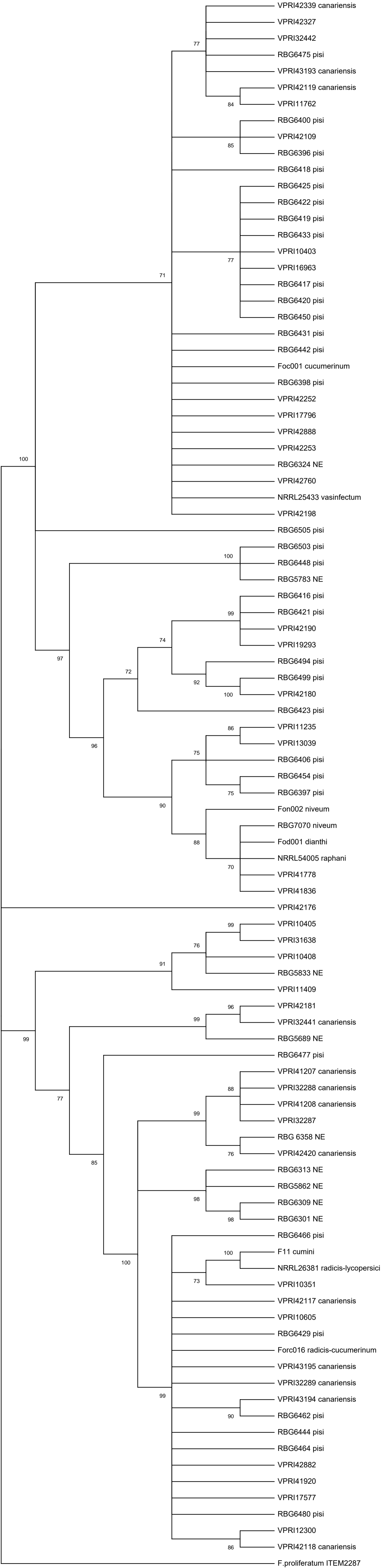

Supplement: Supplementary file 8 — Additional file 8: Supplementary Figure 5. Maximum likelihood consensus tree with bootstrap node support of > 70% was inferred from the sequences of the large variable region of Variant 1 Fusarium oxysporum isolates. Eight reference isolates (Foc001, NRRL25433, Fon002, Fod001, NRRL54005, F11, NRRL26381 and Forc016) from Brankovics et al. [23] were included in the analysis. The tree was rooted to Fusarium proliferatum (ITEM2287). [file 12864_2020_6640_MOESM8_ESM.pdf]

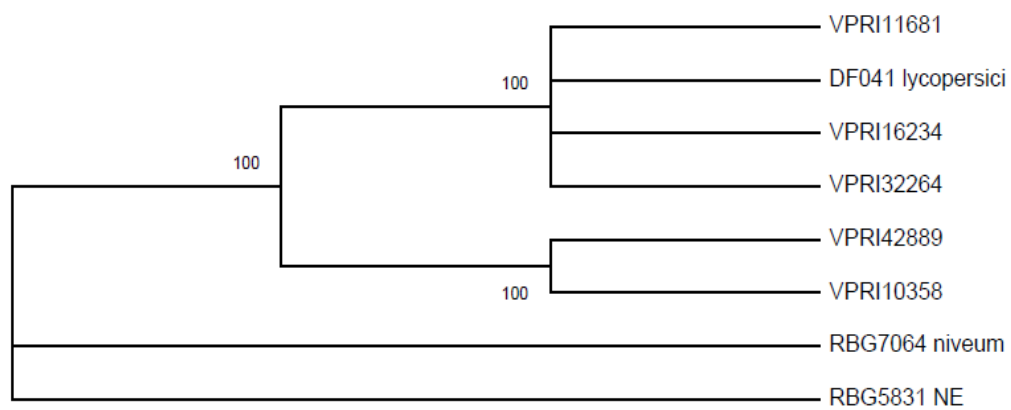

a) Variant 2 isolates

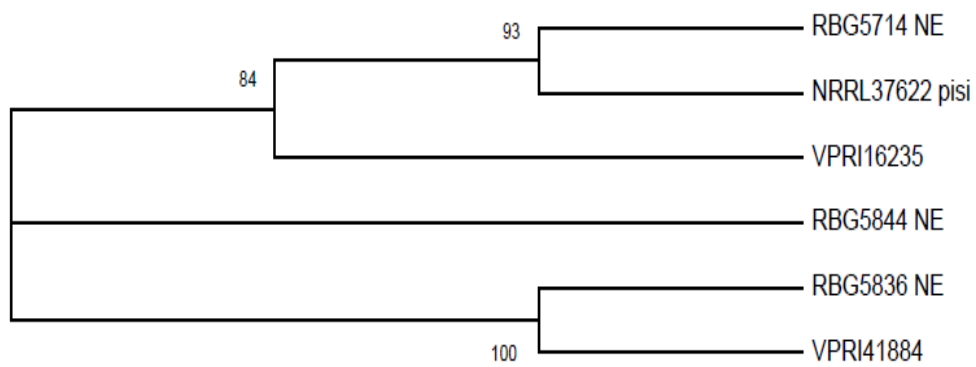

b) Variant 3 isolates

Supplement: Supplementary file 9 — Additional file 9: Supplementary Figure 6. Maximum likelihood consensus tree with bootstrap node support of > 70% was inferred from the sequences of the large variable region of Variant 2 and 3 Fusarium oxysporum isolates. One reference isolate per variant type (V2-DF041, V3-NRRL37622) from Brankovics et al. [23] was included in the analysis. The trees were not rooted. [file 12864_2020_6640_MOESM9_ESM.pdf]

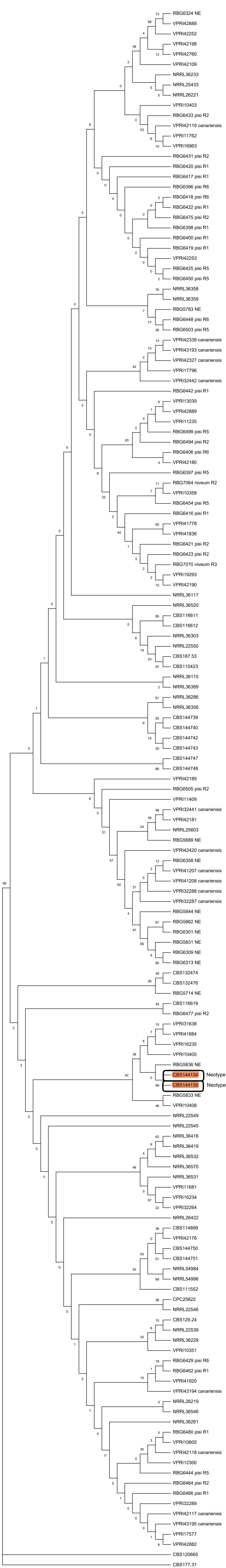

Supplement: Supplementary file 10 — Additional file 10: Supplementary Figure 7. Maximum likelihood tree generated from the combined dataset of Lombard et al. [19] (52 isolates) and isolates from the current study using MEGA X with 1000 bootstrap replications. The analysis was based on concatenated partial gene sequences of cal, RPB2, tef1-α and tub2. Isolates representing the neotype of F. oxysporum are labelled and highlighted in red. Isolates from the current study have the prefixes RBG and VPRI before the numbers. Isolates included from Lombard et al. [19] dataset have prefixes NRRL and CBS before the numbers. The tree was rooted to Fusarium udum (CBS177.31) and Fusarium foetens (CBS120665). [file 12864_2020_6640_MOESM10_ESM.pdf]

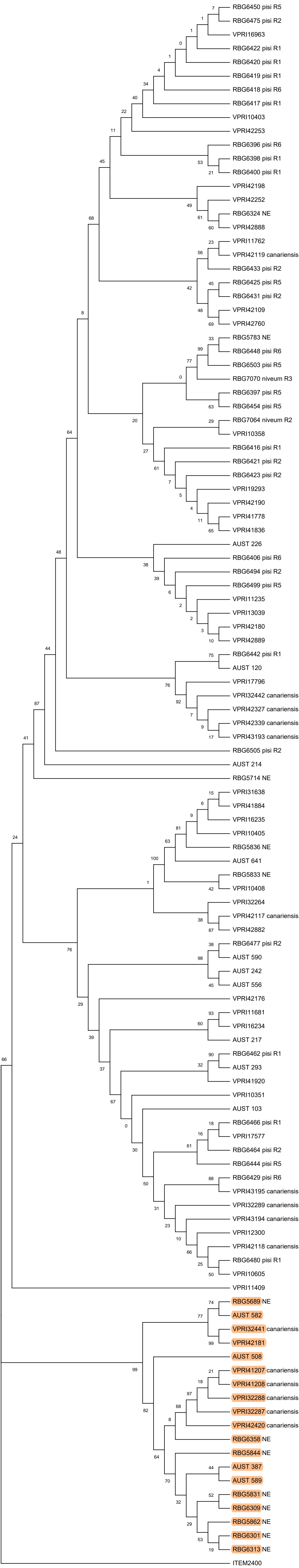

Supplement: Supplementary file 11 — Additional file 11: Supplementary Figure 8. Maximum likelihood tree generated from the combined dataset of Laurence et al. [30] (14 isolates) and isolates from the current study using MEGA X with 1000 bootstrap replications. The analysis was based on concatenated partial gene sequences of mtSSU, RPB1, RPB2, cal, tef1-α, nir, PHO, and acl1. The tree was rooted to Fusarium proliferatum (ITEM2400). Isolates from the current study have the prefixes RBG and VPRI before the numbers while isolates with prefix AUST belong to Laurence et al. [30]. The isolates belonging to Clade 1 (phylogenetic species 1) are highlighted in orange and this is concordant with Clade 1 and species 1 in the current study. [file 12864_2020_6640_MOESM11_ESM.pdf]

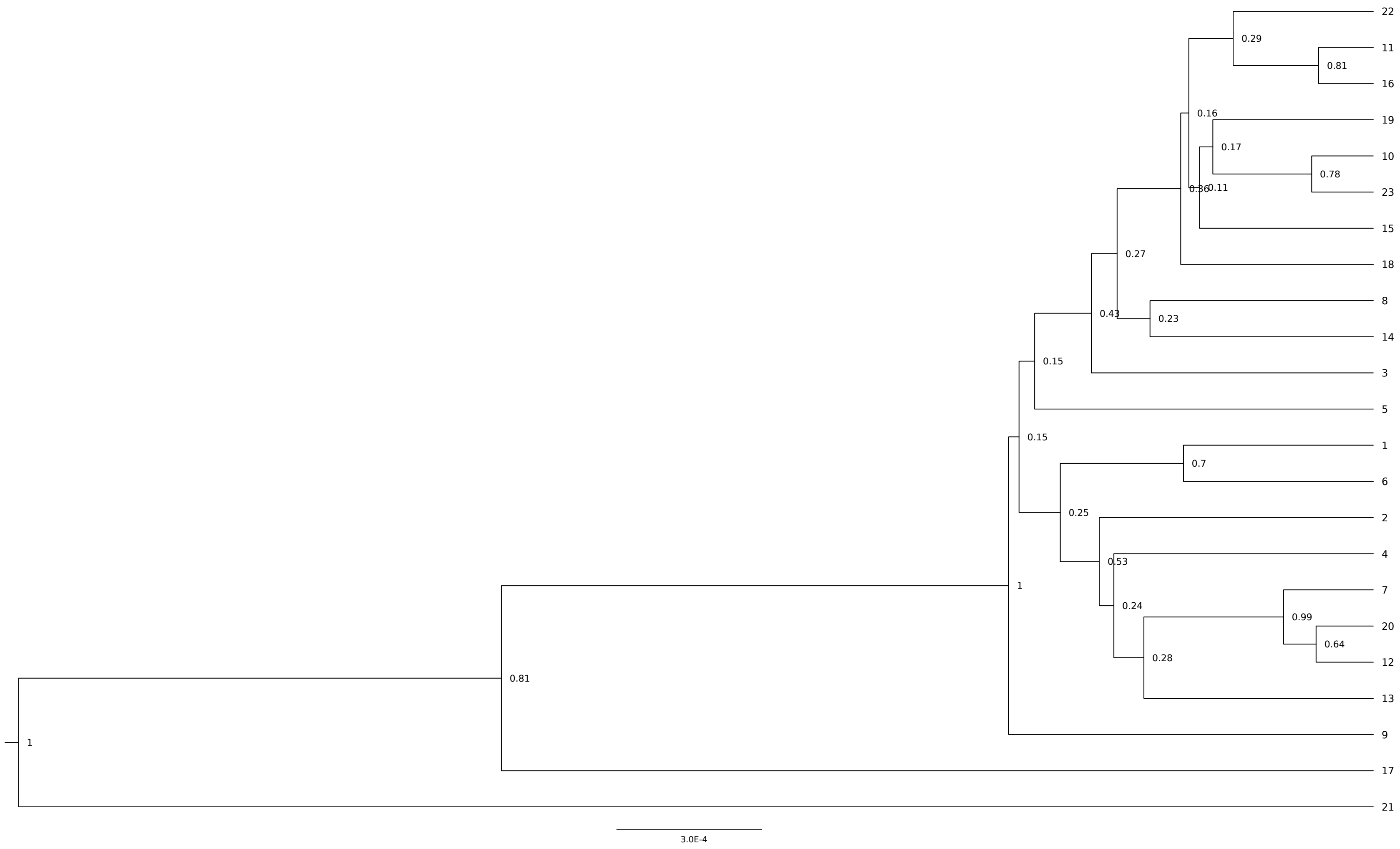

Supplement: Supplementary file 12 — Additional file 12: Supplementary Figure 9. Species tree estimation of Lombard et al. [19] multi-locus DNA sequence dataset using multispecies coalescent (MSC) model in *BEAST. The species denoted by 21, 17 and 9 are F. veterinarium, F. oxysporum and F. foetens respectively. Fusarium foetens is the outgroup. Numbers above branches indicate node support as posterior probabilities. [file 12864_2020_6640_MOESM12_ESM.pdf]
