# Supplementary material for: Phylogenetic relationship between Australian Fusarium oxysporum isolates and resolving the species complex using the multispecies coalescent model
Source: BMC Genomics. 2020 Mar 20;21:248. doi: 10.1186/s12864-020-6640-y (PMC7085163; doi:10.1186/s12864-020-6640-y)

ANOVAOneWay (22/07/2019 22:14:3

One Way ANOVA

Overall ANOVA

	DF	Sum of Squares	Mean Square	F Value	Prob>F
Model	2	5.07601E8	2.538E8	40.84412	5.78218E-14
Error	109	6.77313E8	6213877.9533		
Total	111	1.18491E9			

Null Hypothesis: The means of all levels are equal.
Alternative Hypothesis: The means of one or more levels are different.
At the 0.05 level, the population means are significantly different.

Fit Statistics

	R-Square	Coeff Var	Root MSE	Data Mean
	0.42839	0.05594	2492.76512	44564.76786

Means Comparisons

Fisher Test

	MeanDiff	SEM	t Value	Prob	Alpha	Sig	LCL	UCL
V2 V1	5794.73	974.59511	5.94578	3.36936E-8	0.05	1	3863.11417	7726.34583
V3 V1	-7384.67	1142.32849	-6.46458	2.94799E-9	0.05	1	-9648.72792	-5120.61208
V3 V2	-13179.4	1459.6138	-9.02937	6.97042E-15	0.05	1	-16072.3071	-10286.4929

Sig equals 1 indicates that the difference of the means is significant at the 0.05 level.
Sig equals 0 indicates that the difference of the means is not significant at the 0.05 level.

Means Plot (SD as Error)

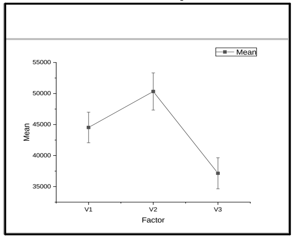

Supplement: Supplementary file 6 — Additional file 6: Supplementary Figure 3. One Way ANOVA and Fisher test for Least Significant Difference between the mitochondrial genome length of different variant types present in the Fusarium oxysporum isolates used for phylogenetic analyses [file 12864_2020_6640_MOESM6_ESM.pdf]
