# Supplementary material for: Phylogenetic relationship between Australian Fusarium oxysporum isolates and resolving the species complex using the multispecies coalescent model
Source: BMC Genomics. 2020 Mar 20;21:248. doi: 10.1186/s12864-020-6640-y (PMC7085163; doi:10.1186/s12864-020-6640-y)

ANOVAOneWay (23/07/2019 09:29:3

One Way ANOVA

Overall ANOVA

	DF	Sum of Squares	Mean Square	F Value	Prob>F
Model	2	1.87568E7	9378389.47357	2.30215	0.10489
Error	109	4.44039E8	4073752.04603		
Total	111	4.62796E8			

Null Hypothesis: The means of all levels are equal.
Alternative Hypothesis: The means of one or more levels are different.
At the 0.05 level, the population means are not significantly different.

Fit Statistics

	R-Square	Coeff Var	Root MSE	Data Mean
	0.04053	0.06133	2018.3538	32911.48214

Means Comparisons

Fisher Test

	MeanDiff	SEM	t Value	Prob	Alpha	Sig	LCL	UCL
V2 V1	-436.66286	789.11476	-0.55336	0.58115	0.05	0	-2000.66265	1127.33694
V3 V1	-1943.12	924.9259	-2.10084	0.03796	0.05	1	-3776.29307	-109.94693
V3 V2	-1506.45714	1181.82697	-1.27469	0.20513	0.05	0	-3848.79978	835.88549

Sig equals 1 indicates that the difference of the means is significant at the 0.05 level.
Sig equals 0 indicates that the difference of the means is not significant at the 0.05 level.

Means Plot (SD as Error)

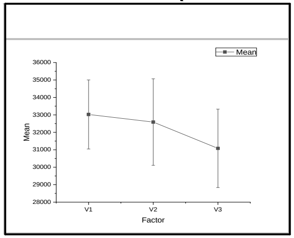

Supplement: Supplementary file 7 — Additional file 7: Supplementary Figure 4. One Way ANOVA and Fisher test for Least Significant Difference between the length of the conserved region of different variant types present in the Fusarium oxysporum isolates used for phylogenetic analyses [file 12864_2020_6640_MOESM7_ESM.pdf]
